# Supplementary material for: Divergent acute versus prolonged pharmacological GLP-1R responses in adult β cell–specific β-arrestin 2 knockout mice
Source: Sci Adv. 2023 May 3;9(18):eadf7737. doi: 10.1126/sciadv.adf7737 (PMC10156113; doi:10.1126/sciadv.adf7737)
Supplement: 20230503-1 [file sciadv.adf7737.v1.pdf]

CELL BIOLOGY

Copyright © 2023 The Authors, some rights reserved; exclusive licensee American Association for the Advancement of Science. No claim to original U.S. Government Works. Distributed under a Creative Commons Attribution License 4.0 (CC BY).

Divergent acute versus prolonged pharmacological GLP-1R responses in adult β cell–specific β-arrestin 2 knockout mice

Stavroula Bitsi[1], Liliane El Eid[1]†, Yusman Manchanda[1]†, Affiong I. Oqua[1], Nimco Mohamed[2], Ben Hansen[2], Kinga Suba[2], Guy A. Rutter[1,3,4], Victoria Salem[2], Ben Jones[5]*, Alejandra Tomas[1]*

The glucagon-like peptide-1 receptor (GLP-1R) is a major type 2 diabetes therapeutic target. Stimulated GLP-1Rs are rapidly desensitized by β-arrestins, scaffolding proteins that not only terminate G protein interactions but also act as independent signaling mediators. Here, we have assessed in vivo glycemic responses to the pharmacological GLP-1R agonist exendin-4 in adult β cell–specific β-arrestin 2 knockout (KO) mice. KOs displayed a sex-dimorphic phenotype consisting of weaker acute responses that improved 6 hours after agonist injection. Similar effects were observed for semaglutide and tirzepatide but not with biased agonist exendin-phe1. Acute cyclic adenosine 5′-monophosphate increases were impaired, but desensitization reduced in KO islets. The former defect was attributed to enhanced β-arrestin 1 and phosphodiesterase 4 activities, while reduced desensitization co-occurred with impaired GLP-1R recycling and lysosomal targeting, increased trans-Golgi network signaling, and reduced GLP-1R ubiquitination. This study has unveiled fundamental aspects of GLP-1R response regulation with direct application to the rational design of GLP-1R–targeting therapeutics.

INTRODUCTION

The glucagon-like peptide-1 receptor (GLP-1R), a class B G protein–coupled receptor (GPCR), is a prominent target for type 2 diabetes (T2D) and obesity treatment. Binding of endogenous GLP-1 to β cell GLP-1Rs potentiates postprandial insulin secretion, with pharmacological agonists successfully leveraging this effect to control blood glucose levels in people with T2D (*1*). GLP-1R agonists (GLP-1RAs) are nonetheless associated with dose-related side effects such as nausea and diarrhea, negatively affecting tolerability, and reducing the range of acceptable dosages (*2*). Exploiting pathway selectivity downstream of GLP-1R activation to potentiate beneficial over detrimental responses has been proposed as a strategy to increase effectiveness, reduce side effects, and improve adherence (*3*–*5*).

The arrestins, a family of cytosolic adaptor proteins, were first identified as key contributors to GPCR homologous desensitization, thereby "arresting" GPCR signaling (*6*, *7*). Later research has assigned them an additional role as bona fide signaling mediators (*8*, *9*), contributing to the concept of "biased agonism," whereby either G protein or arrestin-mediated pathways are preferentially activated. In contrast to arrestins 1 and 4, whose expression is confined to the visual system, β-arrestins 1 and 2 (also known as arrestins 2 and 3, respectively) are ubiquitously expressed (*10*). β-Arrestins are classically associated with receptor internalization via clathrin-coated pits (*11*, *12*); however, we and others have shown that they are dispensable for GLP-1R endocytosis (*13*–*15*). Conversely, there is building evidence that the interaction of GLP-1R and β-arrestins results in autonomous signaling events (*14*, *16*). Pharmacological GLP-1RAs biased away from β-arrestin recruitment exhibit improvements in signaling duration and capacity for sustained insulin secretion (*5*), while agonist-induced cyclic adenosine 5′-monophosphate (cAMP)/protein kinase A signaling is prolonged in β-arrestin 1/2 double–knockout (KO) human embryonic kidney (HEK) 293 cells (*17*), suggesting that β-arrestin deficiency favors sustained GLP-1R action.

The two β-arrestin isoforms result from alternative mRNA splicing (*18*) and, despite sharing structural and functional similarities, often exhibit differential and at times contrasting actions (*19*–*21*). β-Arrestin 1 expression is residual in adult pancreatic islets, with ~50-fold increase in β-arrestin 2 mRNA levels compared to β-arrestin 1 in both human and mouse β cells (*22*, *23*). Previous investigations in whole-body constitutive (*24*) and β cell–specific tamoxifen-inducible β-arrestin 2 KO (*25*) murine models have shown that KO mice display compromised glucose-stimulated insulin secretion and glucose tolerance on high-fat but not on regular chow diet. The latter study attributed these impairments to calcium/calmodulin-dependent protein kinase II–dependent mechanisms. However, the effect of β-arrestin 2 deletion in the control of in vivo GLP-1R responses has not been investigated further than with a single ex vivo experiment in islets extracted from β cell–specific β-arrestin 2 KO lean male mice reporting no significant alterations in acute signaling under these conditions (*25*).

Thus, KO studies suggest that loss of β-arrestin 2 results in deleterious effects on whole-body glucose metabolism (particularly under metabolic stress), with currently unknown effects on pharmacological GLP-1R responses, while modified pharmacological GLP-1RAs with reduced β-arrestin recruitment propensity exhibit improved glucose-lowering potency over prolonged stimulations.

[1]Section of Cell Biology and Functional Genomics, Division of Diabetes, Endocrinology and Metabolism, Department of Metabolism, Digestion and Reproduction, Imperial College London, London, UK. [2]Department of Bioengineering, Imperial College London, London, UK. [3]CHUM Research Centre, Faculty of Medicine, University of Montreal, Quebec H2X 0A9, Canada. [4]Lee Kong Chian School of Medicine, Nanyang Technological University, Singapore 637553, Singapore. [5]Section of Endocrinology and Investigative Medicine, Division of Diabetes, Endocrinology and Metabolism, Department of Metabolism, Digestion and Reproduction, Imperial College London, London, UK.
*Corresponding author. Email: a.tomas-catala@imperial.ac.uk (A.T.); ben.jones@imperial.ac.uk (B.J.)
†These authors contributed equally to this work.

Additional open questions include the role of β-arrestins in the control of GLP-1R trafficking, particularly the degree of their requirement for GLP-1R internalization. Overall, the role of β-arrestin 2 in modulating pharmacological GLP-1R responses in primary β cells, as well as its associated mechanisms, remains poorly characterized.

Given the potential significance of β-arrestin 2 in modulating GLP-1R function, exemplified by the recent success of tirzepatide, a dual GLP-1R/glucose-dependent insulinotropic polypeptide receptor (GIPR) agonist with reduced propensity for β-arrestin recruitment at the GLP-1R (*26*), we have set up here to determine the effect of deleting the β-arrestin 2 gene *ARRB2* specifically from adult pancreatic β cells in acute versus sustained pharmacological GLP-1R responses using a range of in vivo, ex vivo, and in vitro approaches, unveiling a previously unknown dual role of β-arrestin 2 in the control of GLP-1R signaling, with an initial acute potentiating effect that then progresses to a dampening effect over prolonged agonist stimulations. We have additionally identified a compensatory activity of β-arrestin 1 and the cAMP phosphodiesterase 4 (PDE4) that accounts for the acute GLP-1R signaling defect in β-arrestin 2–deleted β cells. Last, we have unveiled changes in GLP-1R postendocytic trafficking and ubiquitination signatures in the absence of β-arrestin 2 that correlate with altered GLP-1R association with the ubiquitin ligase neural precursor cell–expressed, developmentally down-regulated 4 (NEDD4) and GLP-1R signal prolongation.

RESULTS

Deletion of β cell β-arrestin 2 exerts sex- and dose-dependent effects on GLP-1R agonism in vivo

To explore the importance of β-arrestin 2 in the pharmacological GLP-1R responses from adult mice, we generated a β cell–specific, tamoxifen-inducible β-arrestin 2 KO mouse model (Fig. 1A and fig. S1A; Pdx1-Cre-ERT/Barr2$^{fl/fl}$ and control Barr2$^{fl/fl}$ mice). *ARRB2* gene expression, determined by quantitative polymerase chain reaction (qPCR) from whole islets (which contain ~80% β cells), was down-regulated by 67% in KO versus littermate control islets after tamoxifen induction, while β-arrestin 1 gene *ARRB1* levels were not significantly altered (Fig. 1B). The weight, fasting, and fed glycemia of male or female mice on chow diet did not differ between genotypes (fig. S1, B and C). There were also no detectable differences in islet ultrastructure or insulin granule density as assessed by transmission electron microscopy (EM) (fig. S1D), confirming previously reported data (*25*). In addition, we did not observe any significant differences in oral glucose tolerance between the genotypes (fig. S1E), although we did detect a small tendency toward improved insulin sensitivity in β cell–specific β-arrestin 2 KO versus control animals, as assessed by intraperitoneal insulin tolerance test (IPITT), that did not reach statistical significance (fig. S1F).

Noting that the antihyperglycemic effect of GLP-1RAs biased away from β-arrestin recruitment becomes more prominent later into the dosing window (*5*), we performed intraperitoneal glucose tolerance tests (IPGTTs) both acutely and 6 hours after administration of exendin-4 at a range of doses (0.1, 1, and 10 nmol/kg). For lean male mice, we observed improved IPGTT responses in β cell–specific β-arrestin 2 KO versus control animals at 6 hours after administration of exendin-4 (1 nmol/kg), with a similar tendency also present after administration of exendin-4 (0.1 nmol/kg) (Fig. 1, C and D, and fig. S1G). Conversely, KO female mice on chow diet displayed markedly worse acute glycemic responses following exendin-4 (1 nmol/kg) administration, but glucose responsiveness was regained at 6 hours after treatment (Fig. 1E and fig. S1H), leading to significantly reduced Δarea under the curve (ΔAUC; calculated as 6 hours minus acute AUCs as a measure of receptor desensitization) when compared with littermate control female mice (Fig. 1F). Additional studies in female mice further confirmed the β-arrestin 2 KO ΔAUC phenotype observed with exendin-4, an effect that was not present for the same dose of exendin-phe1 (Fig. 1, G and H), an exendin-4 derivative biased away from β-arrestin recruitment (*5*), and hence potentially less reliant on β-arrestin 2 engagement. Concomitantly, and reflecting the results from Fig. 1G, plasma insulin levels measured from samples collected 10 min into the last IPGTT experiments displayed a near-significant tendency to be reduced acutely but increased at 6 hours after exendin-4 treatment for β-arrestin 2 KO versus control mice when normalized to their corresponding vehicle values (fig. S1I), resulting in a highly significant increase in prolonged over acute levels in the β-arrestin 2 KO animals (Fig. 1I).

We next assessed the effect of the β cell–specific β-arrestin 2 deletion on the related glucose-dependent insulinotropic polypeptide (GIP) receptor (GIPR). To this end, we tested the antihyperglycemic effects of the stable GIPR agonist D-Ala2-GIP using similar methodology as above. Similar trends for improved KO responses at 6 hours versus acutely were observed for the GIPR, although they did not reach statistical significance (fig. S2, A to D), suggesting reduced relevance of β-arrestin 2 engagement for the GIPR compared to the GLP-1R. Notably, the exendin-4 effect was not present when using a whole-body, tamoxifen-inducible β-arrestin 2 KO model (R26-Cre-ERT2/Barr2$^{fl/fl}$), a result that we speculate might be linked to the diverse effects of β-arrestin 2 deletion on a range of receptors across different metabolically relevant organs, including GLP-1Rs expressed in different cell types and tissues that might exert compensatory effects on the β cell–specific GLP-1R phenotype (fig. S2, E to H).

Next, to investigate the identified phenotype in a model of diet-induced metabolic stress and T2D, we administered high-fat high-sucrose (HFHS) diet to both control and β cell β-arrestin 2 KO mice. KO males gained more weight compared with control littermates after prolonged (>8-week) HFHS diet exposure (fig. S3A). Fasting glycemia was elevated in HFHS-fed β cell β-arrestin 2 KO males but not in females, with no observed changes in fed glycaemia between genotypes (fig. S3B). In addition, increased β cell mass and average islet sizes were observed in pancreata from KO versus control HFHS-fed animals, with no changes in α cell mass and a trend for the α-to-β cell mass ratio to be reduced (fig. S3, C and D). Study of expression levels of β cell "enriched" and "disallowed" genes (*27*, *28*) revealed that enriched genes such as *MafA* or *Ins2* (with a tendency for *Kcjn11*) were significantly up-regulated in KO animals, while expression of disallowed genes (*Acot7*, *Slc16a1*, and *Ldha*) was not altered (fig. S3E).

As previously described (*25*), HFHS-fed KO animals showed worsening glucose tolerance under vehicle conditions compared to control mice, particularly for males. Consistent with our previous results under lean conditions, there was an acute defect in exendin-4 responses from KO female animals that was overcome at 6 hours after agonist exposure, while for HFHS-fed males that we detected a significantly more pronounced glucose-lowering effect of

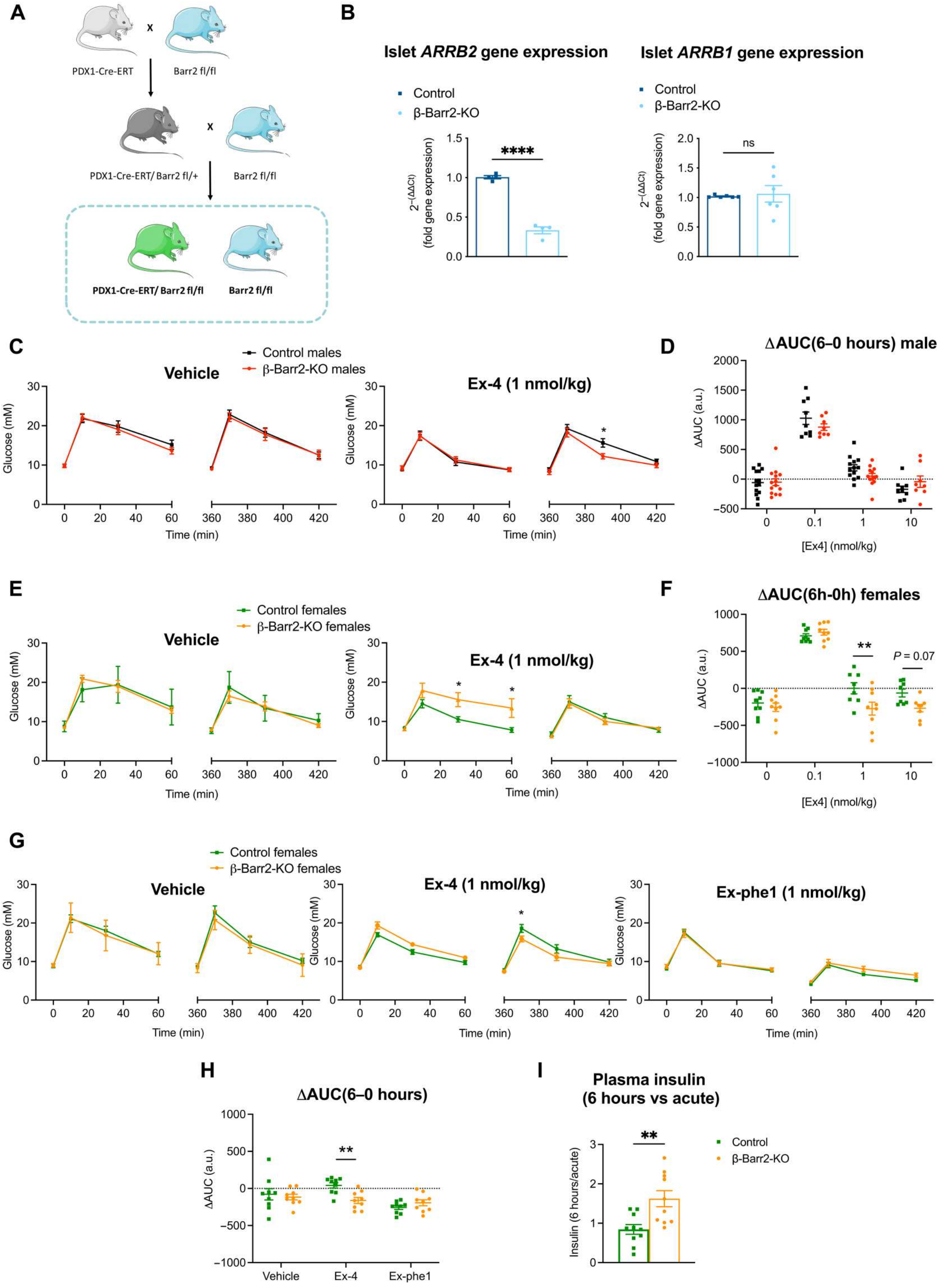

Fig. 1. In vivo GLP-1RA responses in adult β cell–specific β-arrestin 2 KO versus control mice on chow diet. IPGTTs (2 g/kg glucose, intraperitoneally) were performed concurrently with, or 6 hours after intraperitoneal administration of agonists or vehicle (saline). (**A**) Schematic representation of the generation of Pdx1-Cre-ERT Barr2$^{fl/fl}$ and control Barr2$^{fl/fl}$ mice. (**B**) Relative gene expression of β-arrestin 2 (*ARRB2*) ($n = 4$) and β-arrestin 1 (*ARRB1*) ($n = 6$) in adult β cell–specific β-arrestin 2 KO (β-Barr2-KO) versus control mice. (**C**) Glucose curves for vehicle or exendin-4 (Ex-4) administration at 1 nmol/kg in lean, male mice ($n = 13$ to 15 per genotype; age, 12 to 16 weeks). (**D**) Corresponding ΔAUCs (6 to 0 hours) generated from data in (C) and fig. S1G. (**E**) Glucose curves for vehicle or exendin-4 administration at 1 nmol/kg in lean, female mice ($n = 9$ per genotype; age, 12 to 16 weeks). (**F**) Corresponding ΔAUCs (6 to 0 hours) generated from data in (E) and fig. S1H. (**G** and **H**) Glucose curves (G) and corresponding ΔAUCs (6 to 0 hours) (H) for vehicle, exendin-4 (1 nmol/kg), or exendin-phe1 (Ex-phe1; 1 nmol/kg) administration in lean, female mice ($n = 9$ per genotype; age, 14 to 18 weeks). (**I**) Plasma insulin fold changes (6 hours over acute) from control versus KO mice calculated from data in fig. S1I. Comparisons were made with paired or unpaired t tests or two-way analysis of variance (ANOVA) with Sidak's post hoc tests. *$P < 0.05$, **$P < 0.01$, and ****$P < 0.0001$ versus control group. a.u., arbitrary units; ns, not significant. Data are presented as means ± SEM.

exendin-4 after glucose challenge 6 hours after agonist injection with no acute changes (Fig. 2, A and B). Both effects led to similar differences in control versus KO ΔAUCs calculated as above (Fig. 2C), resulting in significantly decreased glucose levels at 6 hours after agonist stimulation when normalized against corresponding vehicle results (Fig. 2D). To interrogate whether these effects would also be elicited by clinically relevant agonists, we performed an analogous study in a mixed sex cohort of HFHS-fed animals with the long-acting GLP-1RA semaglutide and the dual GLP-1R/GIPR agonist tirzepatide [which shows reduced β-arrestin recruitment at the GLP-1R but not at its preferentially binding GIPR (*26*)] and observed a similar profile of improved responses at 72 hours versus 24 hours after agonist injection in KO versus control animals (Fig. 2, E and F), indicating that lack of β-

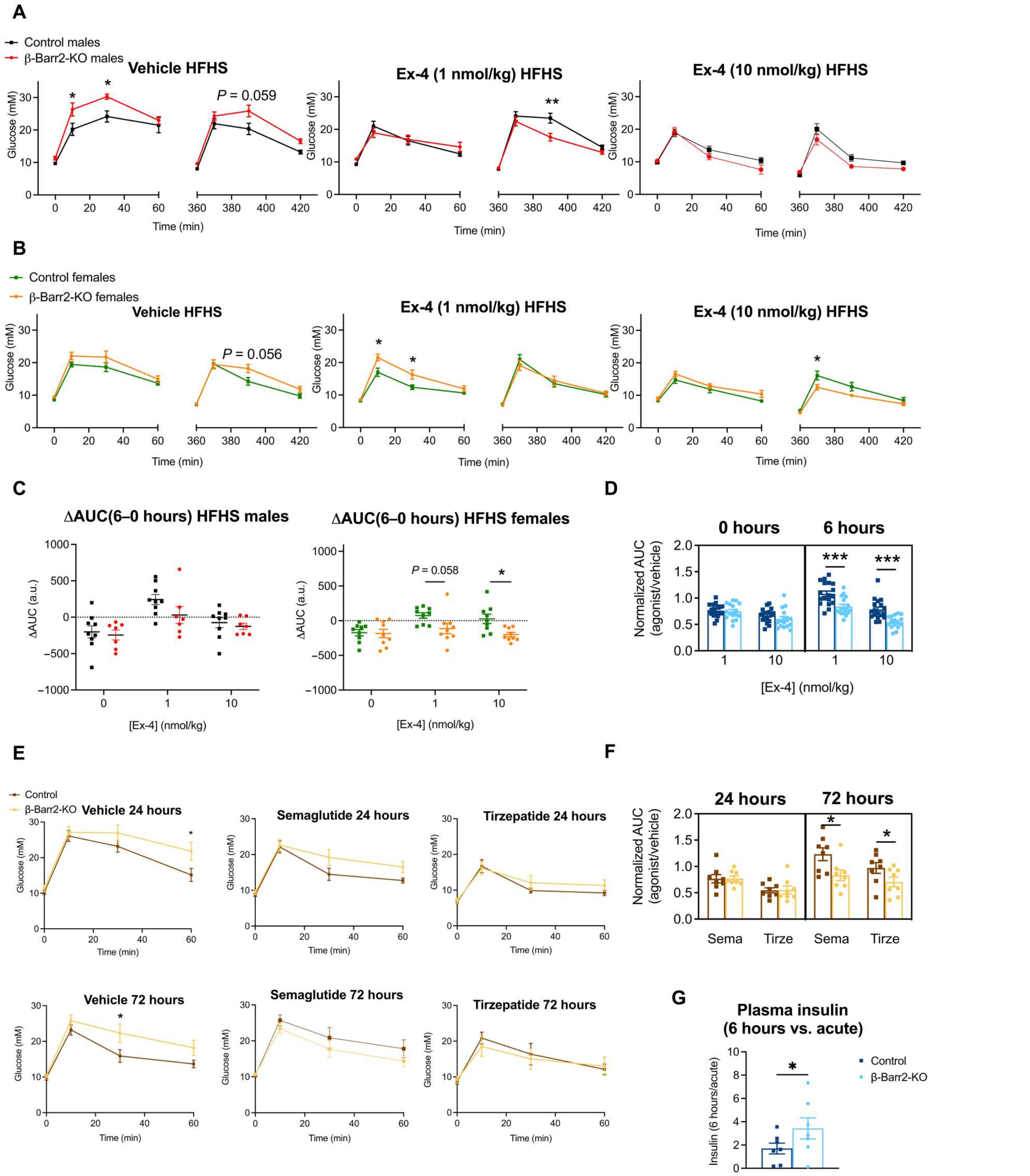

Fig. 2. In vivo GLP-1RA responses in adult β cell–specific β-arrestin 2 KO versus control mice on HFHS diet. (**A**) Glucose curves for vehicle or exendin-4 administration at 1 and 10 nmol/kg in HFHS diet–fed male mice ($n = 7$ to 9 per genotype; duration of HFHS diet, 8 to 12 weeks, initiated at 8 weeks of age). (**B**) Glucose curves for vehicle or exendin-4 administration at 1 and 10 nmol/kg in HFHS diet–fed female mice ($n = 9$ per genotype; duration of HFHS diet, 8 to 12 weeks, initiated at 8 weeks of age). (**C**) ΔAUCs (6 to 0 hours) for male and female HFHS diet–fed mice from (A) and (B). (**D**) Normalized AUCs demonstrating the effect of exendin-4 versus vehicle ($AUC_{Ex\text{-}4}/AUC_{vehicle}$) in HFHS diet–fed mice calculated from combined data presented in (A) to (C). (**E**) Glucose curves for vehicle, semaglutide (10 nmol/kg), or tirzepatide (10 nmol/kg) treatments; IPGTTs performed at 24 or 72 hours after agonist intraperitoneal injection in a mixed sex cohort ($n = 8$ per genotype; duration of HFHS diet, 8 to 12 weeks, initiated at 8 weeks of age). (**F**) Normalized AUCs demonstrating the agonist effect versus vehicle ($AUC_{agonist}/AUC_{vehicle}$) in HFHS diet–fed mice calculated from data presented in (E). (**G**) Plasma insulin fold changes (6 hours over acute) from control versus KO mice calculated from data in fig. S3F. Comparisons were made with unpaired *t* tests, two- or three-way ANOVA with Sidak's post hoc tests. *$P < 0.05$, **$P < 0.01$, and ***$P < 0.001$ versus control group. Data are presented as means ± SEM.

arrestin 2 is beneficial for prolongation of clinically relevant GLP-1RA action in vivo. In addition, and as for chow-fed animals, there was a significant increase in plasma insulin levels assessed 10 min into the IPGTTs from HFHS-fed animals at 6 hours after exendin-4 exposure when normalized to their corresponding acute levels (Fig. 2G and fig. S3F).

β-Arrestin 2 regulates GLP-1R–triggered islet cAMP dynamics

To gain further insight into the mechanism underpinning this contrasting acute versus prolonged glycemic effect, we undertook further investigations in isolated islets from these animals. First, to investigate the role of β-arrestin 2 in the modulation of β cell GLP-1R–dependent cAMP dynamics, we generated a β cell–specific, tamoxifen-inducible mouse line conditionally expressing the *cAMP Encoded Reporter* (*CAMPER*) gene, which encodes the cAMP fluorescence resonance energy transfer (FRET) biosensor ^{T}Epac^{VV} (*29*), exclusively from β cells (Fig. 3A). Time-lapse FRET microscopy experiments in control versus β cell–specific β-arrestin 2 KO *CAMPER* islets revealed significantly reduced acute cAMP responses to exendin-4 in KO versus control animals (Fig. 3B). On the other hand, when islets were pretreated with 1 nM exendin-4 overnight before washing and restimulation with GLP-1 to probe β-arrestin 2 relevance for GLP-1R desensitization, we found that KO islets regained higher cAMP responsiveness when compared with controls, therefore displaying a reversal of the acute cAMP production defect (Fig. 3C).

The cAMP phenotype was also validated in our original non-*CAMPER*–expressing mouse model using a homogeneous time-resolved fluorescence (HTRF) assay in response to a range of exendin-4 concentrations, where we again detected an acute cAMP defect under β cell β-arrestin 2 KO conditions, with Emax and logEC50 parameters reduced in both chow- and HFHS-fed mouse islets (fig. S4, A and B). We additionally performed time-lapse cAMP imaging experiments in a non-FRET system by infecting islets with a baculovirus expressing the cAMP difference detector in situ (cADDis) biosensor (*30*), and, again, in agreement with our *CAMPER* islet results, KO islets displayed a significant defect in acute cAMP versus controls (fig. S4C), but no difference was detected in GLP-1 responses from desensitized islets (fig. S4D).

We next hypothesized that, as the level of β-arrestin 1 is not reduced in β cell–specific β-arrestin 2 KO islets (Fig. 1B) and GLP-1R has been previously shown to recruit β-arrestin 1 and β-arrestin 2 in β cell lines (*14*), compensatory binding to this alternative β-arrestin isoform in the absence of the normally much more abundant β-arrestin 2 might be responsible for the observed deficiency in cAMP production and/or accumulation. To investigate this, we tested the effect of RNA interference (RNAi)–mediated β-arrestin 1 knockdown (KD) in dispersed [to allow good small interfering RNA (siRNA) access] β cell β-arrestin 2 KO versus control *CAMPER* islets. Time-lapse FRET imaging experiments did indeed reveal that β-arrestin 1 silencing restores normal acute cAMP responses to exendin-4 in β-arrestin 2 KO *CAMPER* islets (Fig. 3D), indicating that the previously identified acute cAMP defect is potentially mediated by compensatory β-arrestin 1 binding in the absence of β-arrestin 2. We reasoned that this could result in changes in GLP-1R acute desensitization, including, perhaps, differences in the control of cAMP degradation due to variations in the capacity for PDE recruitment to the receptor between the two different β-arrestin isoforms (*31*). To test this hypothesis and as PDE4 is the dominant PDE isoform in β cells (*32*, *33*), we evaluated the effect of rolipram, a specific PDE4 inhibitor (*34*), on the capacity for cAMP generation from β cell–specific β-arrestin 2 KO and control *CAMPER* islets. The addition of rolipram normalized acute exendin-4–induced cAMP responses in KO islets (Fig. 3E), indicating that changes in PDE4 action underlie the acute cAMP defect of β cell β-arrestin 2 KO islets.

β Cell β-arrestin 2 deletion modifies GLP-1RA–induced intra-islet Ca^{2+} dynamics and connectivity

Changes in intracellular free Ca^{2+} have been described as another downstream signaling readout of GLP-1R activation, controlled mainly by $G\alpha_s$ but also by $G\alpha_q$ protein coupling (*35*). Intracellular calcium rises are more distal within the GLP-1R signaling pathway and linked to insulin granule exocytosis. We used the calcium dye Cal-520 AM and time-lapse fluorescence microscopy to investigate acute intracellular calcium dynamics in response to exendin-4 stimulation in β cell β-arrestin 2 KO versus control islets from either chow- or HFHS-fed animals. Both islet types on chow diet displayed similar exendin-4–induced calcium rises at 6 mM glucose, but the response to a subsequent 11 mM glucose challenge was blunted in KO islets (Fig. 4A). In addition, in keeping with the in vivo observation that HFHS feeding exacerbates the β-arrestin 2 KO phenotype on exendin-4 responses, islets isolated from HFHS-fed KO animals displayed blunted responses to exendin-4 throughout the whole acquisition compared to controls (Fig. 4B).

Next, to investigate calcium responses in vivo, a mouse model of the genetically encoded calcium indicator GCaMP6f (*36*) was crossed with β-arrestin 2 KO mice to generate β cell–specific GCaMP6f-expressing mice that were either β cell β-arrestin 2 KO or wild-type (WT), with islets from these mice subsequently isolated and implanted in the anterior chamber of the eye of WT acceptor mice. This configuration was required to ensure β cell–specific expression of the floxed calcium biosensor in both KOs and WT controls to be used in a platform that supports longitudinal in vivo imaging of calcium responses, as previously shown (*37*, *38*). In vivo calcium experiments were carried out under both chow and HFHS diet conditions, with islet donors and acceptors matched for diet type. Islet calcium "waves" in response to treatment were classified into four activity categories, with one representing the least and four the most active category. In agreement with previous results from diabetic islets (*37*), implanted islets from HFHS-fed mice displayed lower activity compared with their chow diet counterparts (fig. S4E). For chow islets, wave characteristics, including wavelength, amplitude, and full width at half maximum (FWHM), did not differ between treatments and genotypes (fig. S4F). For HFHS diet, however, the amplitude of calcium waves was significantly increased in β-arrestin 2 KO versus control islets at the 6-hour, but not at the 30-min, time point following both vehicle and exendin-4 (10 nmol/kg) administration, while the wavelength and FWHM were not changed (fig. S4G). We also assessed the percentage of connectivity between single cells of an islet, a measure of coordinated intra-islet responses. Connectivity was not significantly different between genotypes after exendin-4 administration on chow diet (fig. S4H); conversely, under HFHS diet conditions, although connectivity initially increased in both genotypes following acute exendin-4 stimulation, this waned over time in control islets from 30 min to 6 hours after exendin-4

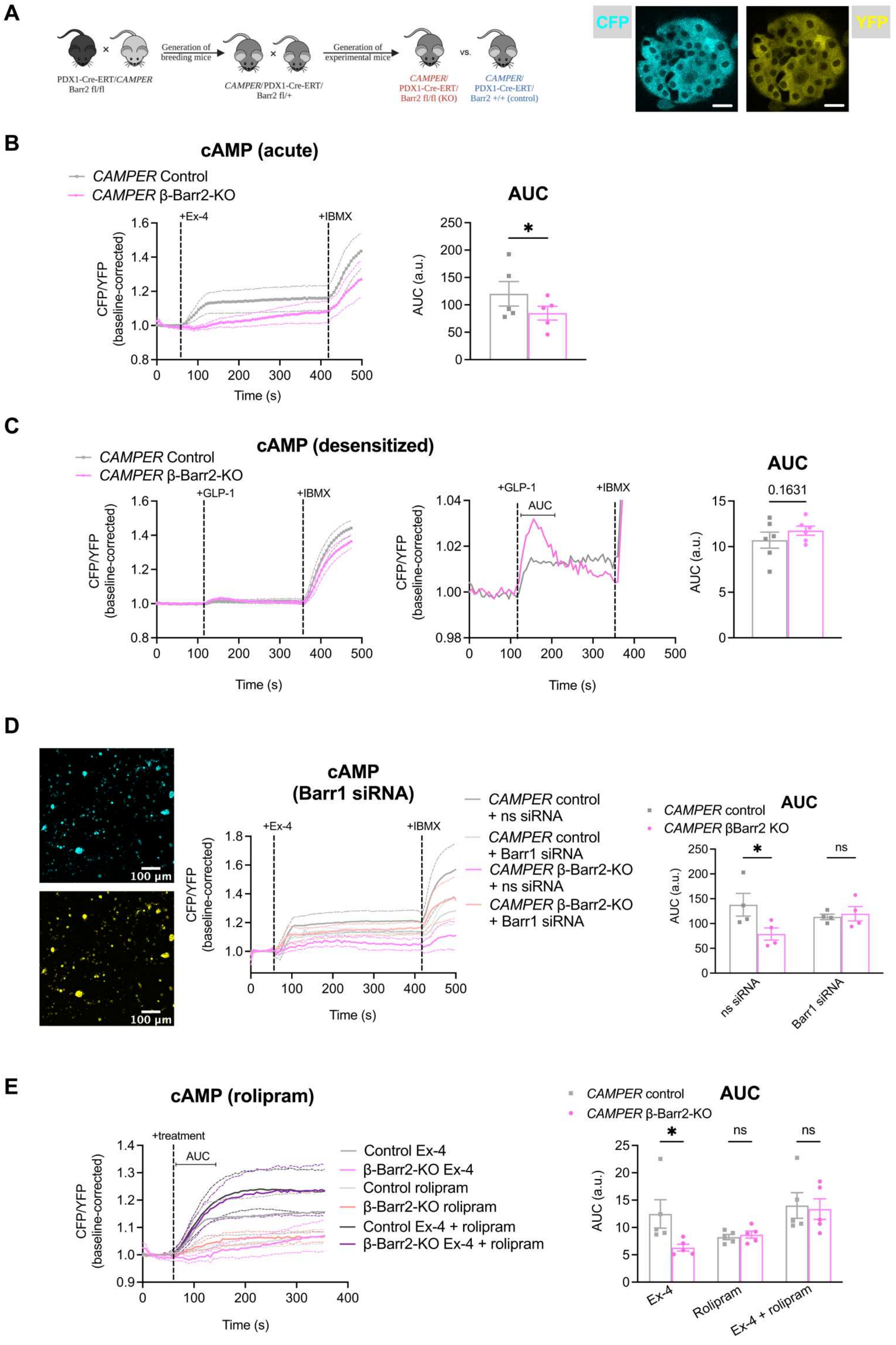

Fig. 3. GLP-1R signaling alterations in adult β cell–specific β-arrestin 2 KO versus control islets. (**A**) Schematic representation of mouse generation and representative images of the cyan fluorescent protein (CFP) and yellow fluorescent protein (YFP) channels from a *CAMPER* islet. Scale bars, 20 μm. (**B**) Changes in FRET (CFP/YFP) over time in isolated β cell–specific β-arrestin 2 KO versus control *CAMPER* islets in response to 100 nM exendin-4, followed by 100 μM isobutyl methylxanthine (IBMX); AUCs calculated for the exendin-4 treatment period (*n* = 5 per genotype). (**C**) Changes in FRET (CFP/YFP) over time in isolated β cell–specific β-arrestin 2 KO versus control *CAMPER* islets pretreated with 1 nM exendin-4 for 16 hours (overnight) in response to 1 nM GLP-1 stimulation, followed by 100 μM IBMX; AUCs calculated for the indicated GLP-1 treatment period (*n* = 6 per genotype). (**D**) Representative images of the CFP and YFP channels and changes in FRET (CFP/YFP) over time in cells from dispersed β cell–specific β-arrestin 2 KO versus control *CAMPER* islets pretreated with nonspecific (ns) control or β-arrestin 1 (Barr1) siRNA for 72 hours in response to 100 nM exendin-4, followed by 100 μM IBMX; AUCs calculated for the exendin-4 treatment period (*n* = 4 per genotype). (**E**) Changes in FRET (CFP/YFP) over time in isolated β cell–specific β-arrestin 2 KO versus control *CAMPER* islets in response to 100 nM exendin-4, 10 μM PDE4 inhibitor rolipram, or a combination of the two; AUCs calculated for the indicated treatment period (*n* = 5 per genotype). Comparisons were made with *t* tests, two-way ANOVA, or mixed-effects model with Sidak's post hoc tests. *$P < 0.05$ versus control group. Data are presented as means ± SEM.

injection, but a higher percentage of connectivity was retained at 6 hours after exendin-4 administration in β-arrestin 2 KO islets (Fig. 4, C and D), suggesting that loss of coordinated calcium responses after sustained exendin-4 exposure is attenuated in vivo by deletion of β-arrestin 2.

We next performed ex vivo insulin secretion assays from isolated islets, with 1-hour exendin-4 incubations used as acute and overnight (16-hour, cumulative) incubations as prolonged readouts. As for cAMP, we again observed reduced acute exendin-4–induced insulin secretion (versus 11 mM glucose) for β cell β-arrestin 2 KO islets from animals on either diet type versus controls. However, this was reversed overnight, with this temporal trajectory conveniently expressed as "overnight over acute," an effect significant for islets from mice on chow diet and close to significance for HFHS diet

Fig. 4. Downstream effects of GLP-1R signaling alterations in adult β cell–specific β-arrestin 2 KO versus control islets. (**A**) Calcium traces and corresponding AUCs and Emax for the indicated treatments in β cell–specific β-arrestin 2 KO versus control islets isolated from animals on chow diet (n = 5 per genotype). (**B**) Calcium traces and corresponding AUCs and Emax for indicated treatments in β cell–specific β-arrestin 2 KO versus control islets isolated from animals on HFHS diet (n = 4 per genotype). (**C**) Cartesian connectivity maps for exemplar islet calcium responses imaged after implantation in the anterior chamber of the eye of WT recipients on HFHS diet, revealing β cell connectivity for β cell–specific β-arrestin 2 KO versus control GCaMP6f islets from HFHS diet donors measured as the R value between each β cell pair; lines are color-coded by R value (see scale). Maps shown at 30-min and 6-hour time points after the recipient mouse had received glucose (2 g/kg) intraperitoneally with either exendin-4 (10 nmol/kg) or saline (vehicle). (**D**) Average connectivity values (average R value for all β cell pairs within an islet) across β cell–specific β-arrestin 2 KO versus control GCaMP6f islets (n = 6 to 8 per genotype) from the experiment described in (C). (**E** and **F**) Insulin secretion fold changes [overnight (O/N) over acute (1 hour)] from control versus β cell–specific β-arrestin 2 KO islets on chow (E) or HFHS diet (F) calculated from data in fig. S4 (I and J) for the indicated agonist. Comparisons between two experimental groups were made with t tests, one- or two-way ANOVA, or mixed-effects model with Sidak's post hoc tests. *P < 0.05 and **P < 0.01. Data are presented as means ± SEM.

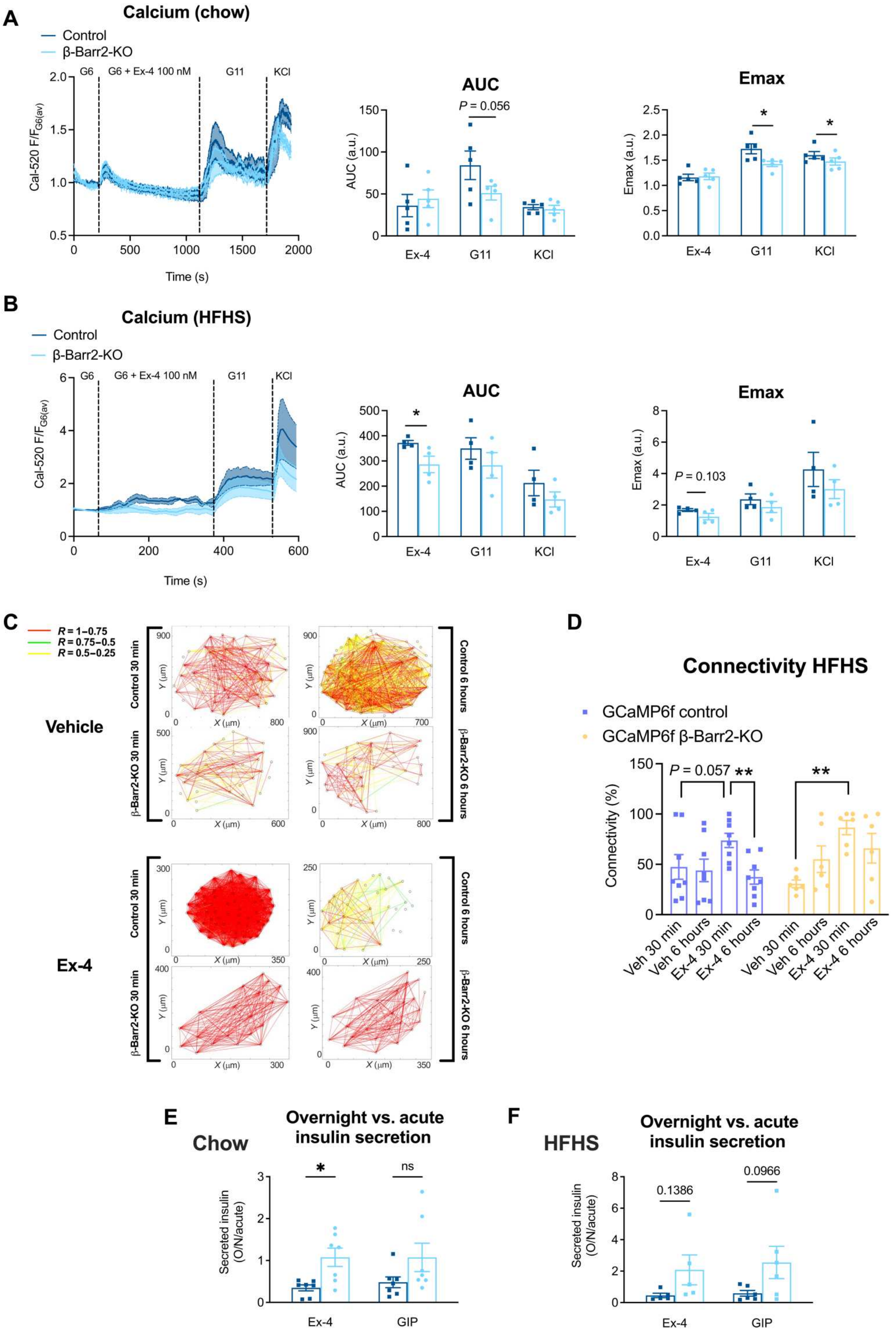

(Fig. 4, E and F, and fig. S4, I and J). Parallel experiments, depicted in the same figure, were performed using the GIPR agonist GIP, where we observed the same trend, which, in agreement with our in vivo results suggesting a lesser dependence of GIPR on β-arrestin 2, did not quite reach statistical significance.

Islet GLP-1R trafficking is perturbed following β-arrestin 2 deletion

We next assessed the potential contribution from alterations in GLP-1R trafficking to the changes in exendin-4–induced signaling in the absence of β-arrestin 2. First, to control for potential differences due to changes in basal cell surface GLP-1R expression, we quantified surface GLP-1R levels from control and β cell β-arrestin

2 KO islets by labeling these with the fluorescent antagonist exendin-9–tetramethylrhodamine (TMR), with no differences observed between the two genotypes (Fig. 5A). We noted that islets of both genotypes from HFHS-fed mice had generally lower exendin-9–TMR binding capacity than chow islets, suggesting reduced GLP-1R surface expression under these conditions. We next determined endogenous GLP-1R trafficking profiles in β cell β-arrestin 2 KO versus control islets using the previously characterized fluorescent agonist exendin-4–TMR (*39*) as a proxy for GLP-1R localization. We quantified GLP-1R internalization in response to 1-hour exendin-4–TMR exposure by measuring TMR fluorescence levels, using acetic acid buffer wash treatment to strip any remaining exendin-4–TMR bound to noninternalized/cell surface receptors, both for chow- and for HFHS islets (Fig. 5B and fig.

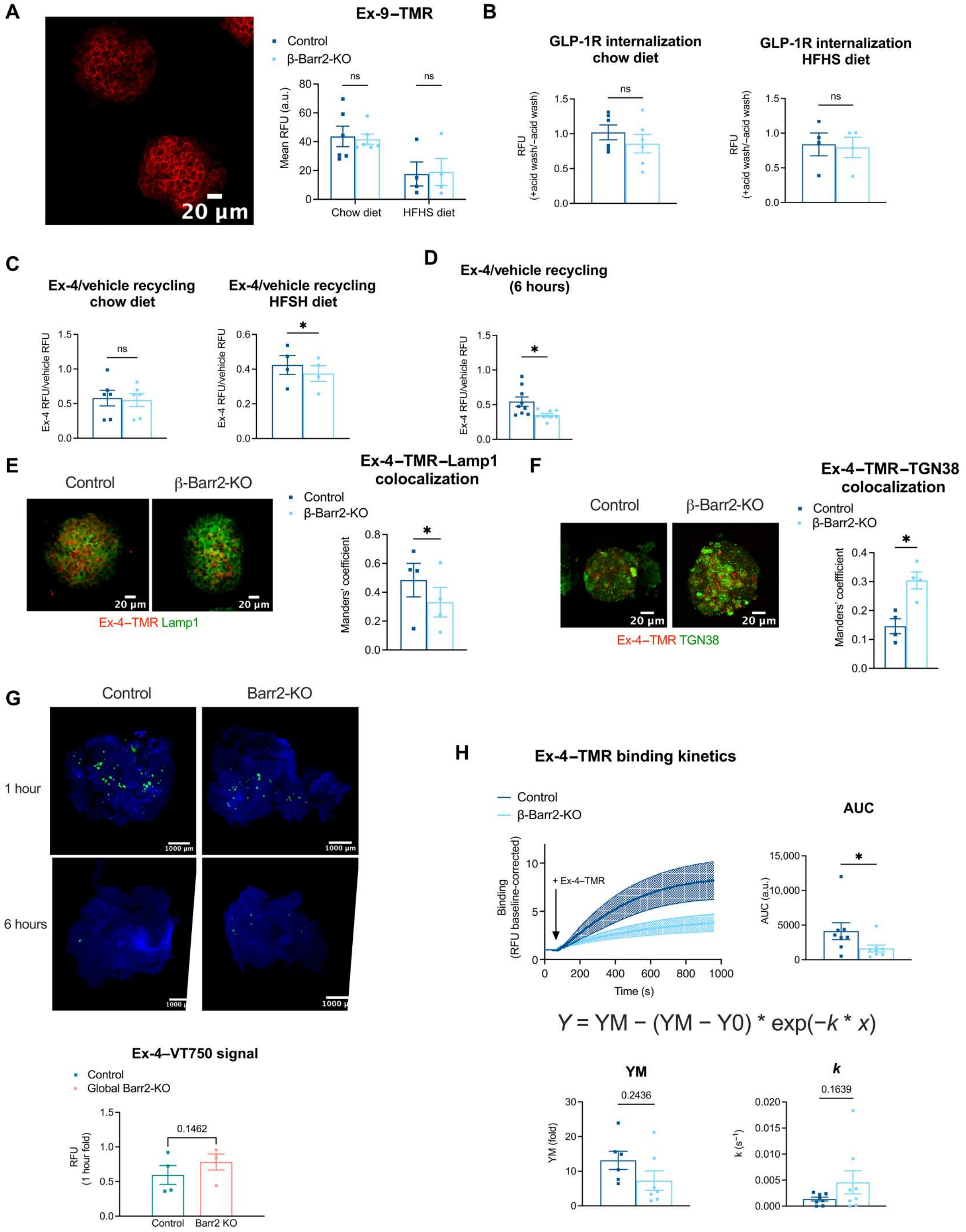

Fig. 5. GLP-1R trafficking in adult β cell–specific β-arrestin 2 KO versus control islets. (**A**) Representative images and fluorescence in β cell–specific β-arrestin 2 KO versus control islets treated with 100 nM exendin-9–TMR for 30 min in chow-fed (*n* = 6 per genotype) or HFHS-fed (*n* = 4 per genotype) mice. (**B**) Exendin-4–TMR fluorescence at 1 hour (fold ± acid wash), as surrogate measure for GLP-1R internalization in β cell–specific β-arrestin 2 KO versus control islets from chow- and HFHS-fed mice, from fig. S5A data. (**C**) Normalized exendin-4 over vehicle GLP-1R 3-hour recycling in β cell–specific β-arrestin 2 KO versus control islets from chow- and HFHS-fed mice, from fig. S5B data. (**D**) Normalized exendin-4 over vehicle GLP-1R 6-hour recycling in β cell–specific β-arrestin 2 KO versus control islets from chow-fed mice, from fig. S5C data. (**E**) Representative images of β cell–specific β-arrestin 2 KO versus control islets labeled for Lamp1 (green) after treatment with 100 nM exendin-4–TMR (red) for 3 hours. Manders' coefficient of colocalization for exendin-4–TMR with Lamp1 is shown (*n* = 4 per genotype). (**F**) Representative images of β cell–specific β-arrestin 2 KO versus control islets labeled for TGN38 (green) after treatment with 100 nM exendin-4–TMR (red) for 3 hours. Manders' coefficient (exendin-4–TMR with TGN38) is shown (*n* = 4 per genotype). (**G**) Representative images from OPT data of pancreata from whole-body β-arrestin 2 KO (Barr2-KO) and control animals injected intraperitoneally with exendin-4–VT750 (100 nmol/kg) 1 hour or 6 hours after injection. Mean exendin-4–VT750 fluorescence (6 hours over 1 hour) is shown for each genotype (*n* = 4 per genotype). (**H**) GLP-1R binding affinity to 100 nM exendin-4–TMR in β cell–specific β-arrestin 2 KO versus control islets pretreated with metabolic inhibitors (*n* = 8 per genotype). AUC, maximum value (YM), and rate constant (*k*) from fitted curves depicted for each genotype. Comparisons were made using *t* tests or two-way ANOVA with Sidak's post hoc tests; *$P < 0.05$ versus control group. Data are means ± SEM. RFU, relative fluorescence units.

S5A). We were unable to detect any differences in GLP-1R internalization between KO and control islets, indicating that, as we had previously observed in β cell lines (*13*), β-arrestin 2 does not play a relevant role in GLP-1R endocytosis.

Next, we assessed the propensity for GLP-1R recycling back to the plasma membrane by incubating control or KO islets with exendin-4–TMR for 3 hours following a prior 1-hour stimulation with unlabeled exendin-4 to trigger maximal receptor internalization in both chow- and HFHS-fed mouse islets. In this assay, reemergence of agonist-internalized GLP-1R at the cell surface is detected by subsequent binding and reuptake of fluorescently labeled exendin-4–TMR. While no differences in GLP-1R recycling were detected in chow islets, HFHS KO islets displayed a significant reduction in GLP-1R recycling following exendin-4 stimulation (Fig. 5C and fig. S5B). We repeated the recycling assay in chow islets with a 6-hour period of receptor recycling to assess any possible changes that might only become apparent at longer time points (Fig. 5D and fig. S5C), and we now also detected a significant reduction in GLP-1R recycling in KO versus control islets. We also assessed the degree of colocalization between exendin-4–TMR and lysosomal-associated membrane protein 1 (LAMP1), a lysosomal marker, as well as TGN38, a marker for the trans-Golgi network (TGN), in intact islets after 3 hours of exendin-4–TMR stimulation (Fig. 5, E and F). Here, we found significantly reduced colocalization between fluorescent exendin-4 and LAMP1 but conversely increased colocalization with TGN38 in KO versus control islets, suggesting reduced capacity for GLP-1R trafficking to lysosomal compartments and redirection to the TGN in the absence of β-arrestin 2.

Next, to assess in vivo exendin-4 accumulation within the pancreas, we performed three-dimensional (3D) optical projection tomography (OPT) imaging and signal quantification from optically cleared whole pancreata extracted from β-arrestin 2 KO or control mice previously injected with the near-infrared exendin-4–derivative exendin-4–VivoTag 750 before intracardial fixation and pancreas extraction 1 hour versus 6 hours after injection (Fig. 5G). Results showed a tendency toward increased fluorescent signal retention in β-arrestin 2 KO compared to control pancreata at 6-hour over 1-hour periods.

Last, we evaluated the GLP-1R capacity for binding to exendin-4–TMR in islets from β cell–specific β-arrestin 2 KO versus control mice imaged by time-lapse confocal microscopy in the presence of metabolic inhibitors to prevent receptor endocytosis (Fig. 5H). Fitted curve results indicated that while kinetic parameters were not altered, there was a significant reduction in β cell β-arrestin 2 KO AUCs compared with control islets, indicating a defect in GLP-1R exendin-4–TMR binding capacity, a phenotype that might contribute to the observed deficit in acute GLP-1R signaling from these islets.

In vitro impact of β-arrestin 2 deletion on GLP-1R trafficking and signaling in a β cell model

After characterization of GLP-1R responses in adult β cell β-arrestin 2 KO mice and primary islets, we next generated an in vitro β cell model for a more detailed examination of molecular mechanisms associated with the changes observed in GLP-1R trafficking and signaling. To validate our model, namely INS-1 832/3 rat insulinoma cells, we first verified plasma membrane recruitment of β-arrestin 2 following GLP-1R stimulation. Time-lapse spinning disk microscopy experiments in INS-1 832/3 cells stably expressing soluble *N*-ethylmaleimide–sensitive factor attachment protein (SNAP)–tagged human GLP-1R transiently transfected with a β-arrestin 2–green fluorescent protein (GFP) construct demonstrated that β-arrestin 2 is recruited from the cytoplasm to the plasma membrane, where it colocalizes with SNAP-GLP-1R within 5 min of exendin-4 stimulation (fig. S6, A and B, and movie S1).

Next, we generated a lentiviral CRISPR-Cas9–derived INS-1 832/3 cell subline in which the *ARRB2* gene was ablated. The resulting KD cells displayed a 51% reduction in β-arrestin 2 expression compared with control cells generated in parallel with a nontargeting construct (Fig. 6A). Using this cell model, we assessed the degree of GLP-1R plasma membrane versus endosomal signaling by implementing a NanoBiT assay based on the Nb37 (*40*), a nanobody that specifically binds to active $G\alpha_s$ proteins following GPCR stimulation (*41*). In agreement with our previously detected acute cAMP defect in β cell β-arrestin 2 KO islets, we measured a significant reduction in Emax, with no change in logEC50, for exendin-4–induced GLP-1R plasma membrane signaling in β-arrestin 2 KD compared with control cells, with no changes in the degree of endosomal signaling (Fig. 6, B and C). A similar experiment performed with the GIPR agonist D-Ala2-GIP in the same cells did not reveal any significant changes in GIPR plasma membrane or endosomal signaling in β-arrestin 2 KD versus control cells (fig. S7, A and B).

Following our ex vivo trafficking results showing reduced GLP-1R targeting to lysosomal compartments but increased TGN rerouting in β-arrestin 2 KO islets, we next assessed the level of signaling specifically from this latter compartment using time-lapse FRET microscopy with a $^{T}Epac^{VV}$-based cAMP biosensor–modified in-house to localize specifically to the TGN (Fig. 6D). Quantification of cAMP production after exendin-4 stimulation with both global (cytoplasmic) and TGN-targeted $^{T}Epac^{VV}$ biosensors demonstrated a significant increase in TGN over global cAMP levels in β-arrestin 2 KD versus control cells (Fig. 6, E and F). Downstream signal transmission was next assessed by Western blot analysis of extracellular signal–regulated kinase 1/2 (ERK1/2) and cAMP response element–binding protein (CREB) phosphorylation in both cell subtypes following different times of exendin-4 exposure. This experiment showed increased basal phospho-ERK1/2 (and a similar trend for basal phospho-CREB) but reduced ERK1/2 and CREB phosphorylation fold increases at 5 min after exendin-4 stimulation in β-arrestin 2 KD versus control cells (Fig. 6, G and H, and fig. S7C).

We next evaluated the endogenous GLP-1R cell surface levels in both cell lines by labeling them with exendin-9–TMR, and, as for primary islets, we found no differences in surface GLP-1R levels between control and β-arrestin 2 KD (fig. S7D). We then performed a comprehensive assessment of GLP-1R trafficking in these cells by using NanoBRET subcellular localization assays based on expression of specific Rab guanosine triphosphatase bystanders at different intracellular locations (fig. S7E), revealing no differences in GLP-1R plasma membrane or Rab5-positive early endosome localization in response to increasing exendin-4 concentrations, but, in agreement with our primary islet results, a significant decrease in GLP-1R localization to Rab11-positive recycling endosomes, associated with reduced Emax values in β-arrestin 2 KD compared with control cells (Fig. 7, A and B).

We also investigated differences in GLP-1R localization to either Rab4- or Rab9-positive compartments, highlighting a fast recycling route or the retrograde endosome-to-Golgi transport, respectively,

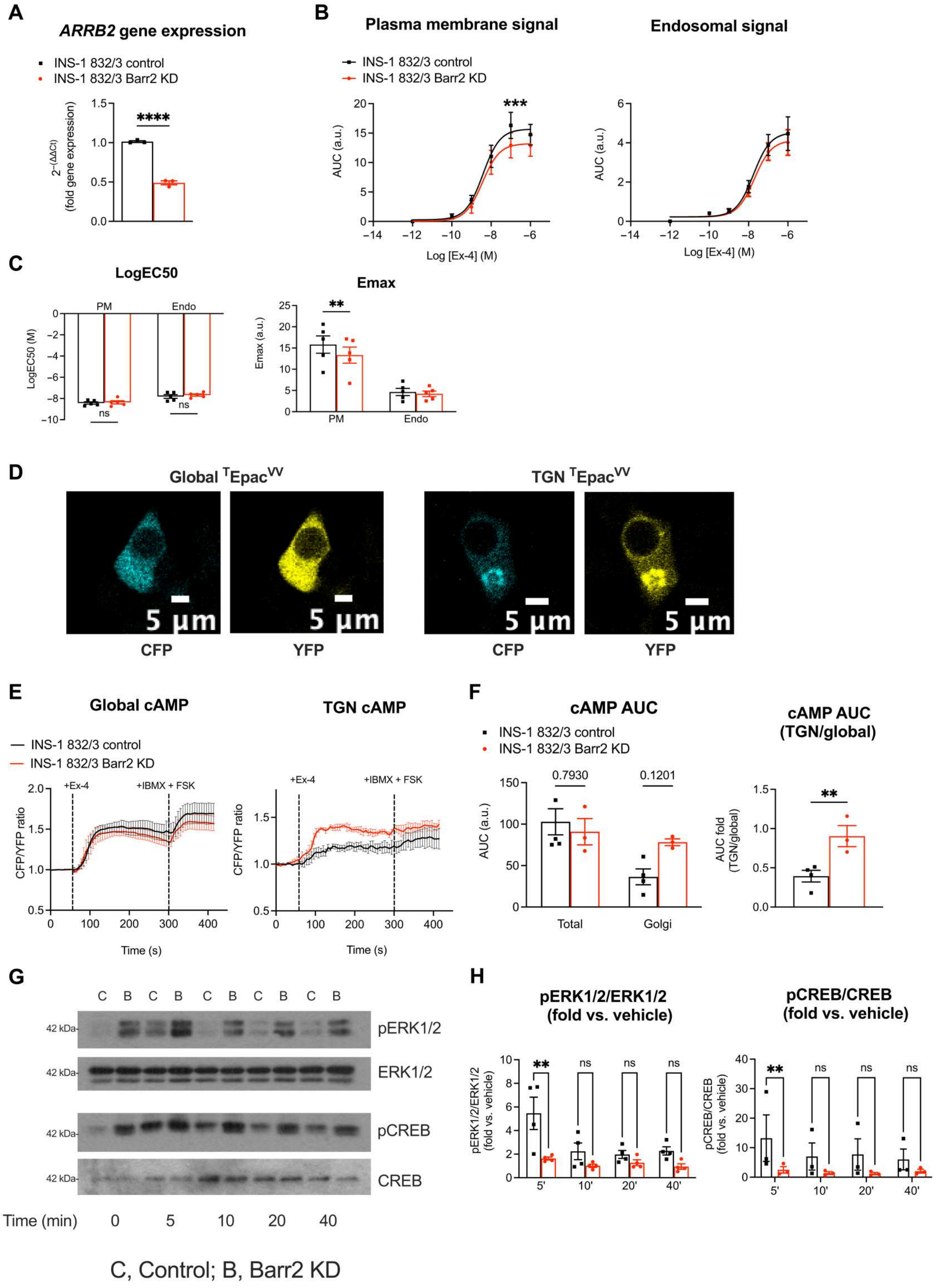

Fig. 6. Spatiotemporal signaling profiles of INS-1 832/3 β-arrestin 2 KD versus control β cells. (**A**) Relative *ARRB2* gene expression in β-arrestin 2 (Barr2) KD versus control INS-1 832/3 cells ($n = 3$). (**B**) Exendin-4 dose-response AUC curves for Nb37-SmBiT and CAAX-LgBiT (plasma membrane) or Endofin-LgBiT (endosomal) signal complementation assays ($n = 5$). (**C**) LogEC50 and Emax values calculated from (B). (**D**) Representative images of CFP and YFP channels from INS-1 832/3 cells transfected either with global (cytoplasmic) or TGN-targeted ^{T}Epac^{VV} cAMP biosensor constructs. (**E**) Changes in FRET (CFP/YFP) over time in INS-1 832/3 Barr2 KD versus control cells expressing global or TGN-targeted ^{T}Epac^{VV} cAMP biosensor constructs in response to 100 nM exendin-4, followed by 100 μM IBMX + 10 μM forskolin ($n = 3$ to 5). (**F**) AUCs calculated for the exendin-4 treatment period from (E). TGN-localized over global cAMP AUCs are depicted for each cell type. (**G**) Representative blots for pERK1/2, total ERK1/2, pCREB, and total CREB in Barr2 KD and control INS-1 832/3 cells after treatment with 100 nM exendin-4 for the indicated time points. (**H**) Quantification of pERK1/2 over total ERK1/2 and pCREB over total CREB (fold versus vehicle) using densitometry analysis ($n = 4$ for pERK1/2 and $n = 3$ for pCREB). Data from fig. S7C were normalized to vehicle levels for each cell type. Comparisons were made using t tests or two-way ANOVA with Sidak's post hoc tests. $^{**}P < 0.01$, $^{***}P < 0.001$, and $^{****}P < 0.0001$ versus control group. Data are presented as means ± SEM.

and while we found no effect on GLP-1R localization to Rab4 endosomes, the GLP-1R recruitment to Rab9-positive compartments was significantly increased in β-arrestin 2 KD cells (Fig. 7C), suggesting that these cells recapitulate the trafficking phenotype obtained in primary islets, with increased GLP-1R rerouting to the TGN. We next performed GLP-1R lysosomal localization studies in control and β-arrestin 2 KD cells transiently expressing SNAP–GLP-1R labeled with a fluorescent SNAP-Surface probe and LysoTracker under vehicle and exendin-4–stimulated conditions (Fig. 7, D and E) and, as for islets, found significantly reduced lysosomal targeting of exendin-4–stimulated GLP-1Rs in β-arrestin 2 KD versus control cells.

Last, we investigated the degree of GLP-1R ubiquitination as a potential mechanism to explain the postendocytic trafficking

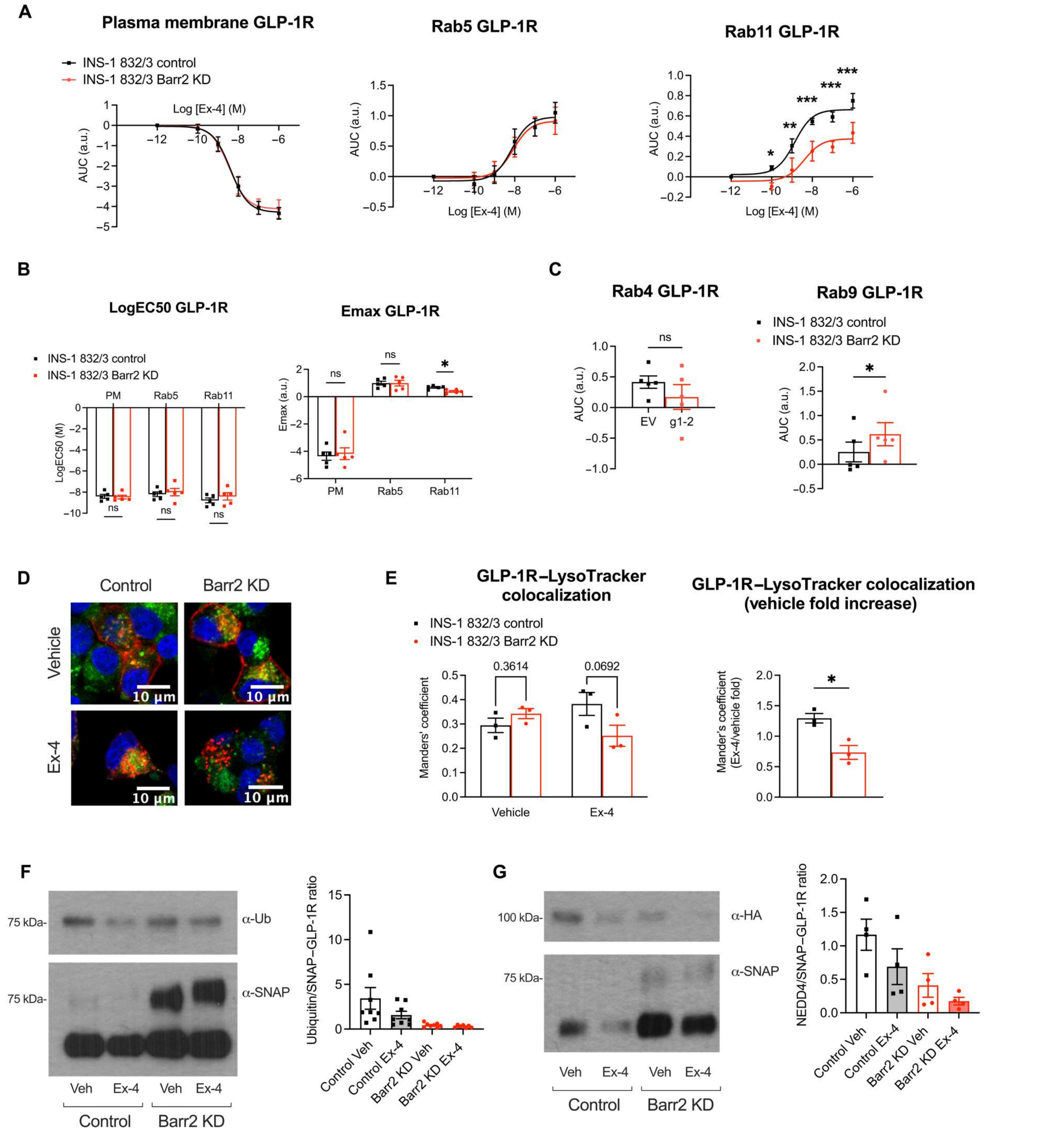

Fig. 7. Associated changes in GLP-1R trafficking and ubiquitination in INS-1 832/3 β-arrestin 2 KD versus control β cells. (**A**) Exendin-4 dose-response AUC curves for SNAP–GLP-1R–NLuc and KRAS-Venus (plasma membrane), Rab5-Venus (early endosomes), and Rab11-Venus (recycling endosomes) NanoBRET assays ($n = 5$). (**B**) LogEC50 and Emax values calculated from (A). (**C**) AUC for NanoBRET responses to 100 nM exendin-4 using SNAP–GLP-1R–NLuc and Rab4-Venus (fast recycling endosomes) or Rab9-Venus (endosome-to-TGN). (**D**) Representative images of SNAP–GLP-1R–expressing Barr2 KD and control cells labeled with SNAP-Surface 647 (red) and treated with 100 nM exendin-4 or vehicle for 3 hours and 1 μM LysoTracker Red DND-99 (green) for the last 5 min of the incubation period to label the lysosomes; nuclei, 4′,6-diamidino-2-phenylindole (blue). (**E**) Manders' coefficient of colocalization for SNAP–GLP-1R with LysoTracker Red DND-99 in INS-1 832/3 Barr2 KD versus control cells ($n = 3$). Exendin-4 over vehicle fold values depicted as a surrogate measure of agonist-induced SNAP–GLP-1R degradation ($n = 3$). (**F**) Representative blots showing SNAP-GLP-1R and corresponding ubiquitination (Ub) levels following SNAP–GLP-1R immunoprecipitation from INS-1 832/3 SNAP-GLP-1R Barr2 KD versus control cells under vehicle conditions of after stimulation with 100 nM exendin-4 for 10 min, with quantification of ubiquitin over SNAP levels for the different conditions shown ($n = 8$). (**G**) Representative blots showing SNAP-GLP-1R and HA-NEDD4 levels following SNAP-GLP-1R immunoprecipitation from INS-1 832/3 SNAP-GLP-1R Barr2 KD versus control cells under vehicle conditions of after stimulation with 100 nM exendin-4 for 10 min, with quantification of HA over SNAP levels for the different conditions shown ($n = 4$). Comparisons were performed using *t* tests or one- or two-way ANOVA with Sidak's post hoc tests. *$P < 0.05$, **$P < 0.01$, and ***$P < 0.001$. Data are presented as means ± SEM.

differences observed between β-arrestin 2 KD and control cells. For this, we performed GLP-1R immunoprecipitation experiments in INS-1 832/3 control and β-arrestin 2 KD cells stably expressing SNAP/FLAG-tagged human GLP-1R (Fig. 7F and fig. S7F). Results in control cells showed that the GLP-1R is constitutively ubiquitinated under vehicle conditions and subsequently undergoes partial deubiquitination following exendin-4 stimulation. They also revealed a pronounced reduction in GLP-1R ubiquitination in β-arrestin 2 KD cells, present under both vehicle and stimulated conditions, so that further deubiquitination in response to exendin-4 is no longer significant in these cells. We have previously identified, in an RNAi screen for factors involved in regulating exendin-4–stimulated insulin secretion (*42*), the E3 ubiquitin ligase NEDD4, best known to mediate β-arrestin 2–dependent ubiquitination and endolysosomal sorting of GPCRs following ligand stimulation (*43*, *44*), as a factor involved in the maintenance of exendin-4–stimulated insulin secretion. More recently, we have identified NEDD4 in a list of potential GLP-1R interactors during mass spectrometry analysis of the receptor interactome in INS-1 832/3 cells. We therefore analyzed whether the changes in GLP-1R ubiquitination detected in cells without β-arrestin 2 could be attributed to altered NEDD4 recruitment to the receptor. To test this, we coimmunoprecipitated NEDD4 with GLP-1R from control and β-arrestin 2 KD cells stably expressing SNAP/FLAG–GLP-1R and transiently transfected with a hemagglutinin (HA)–tagged NEDD4 construct to quantify the level of HA-NEDD4-SNAP:-FLAG–GLP-1R association under vehicle versus exendin-4–stimulated conditions (Fig. 7G and fig. S7G). Results closely correlated with those obtained for GLP-1R ubiquitination, with GLP-1R constitutively associated with NEDD4, and GLP-1R:NEDD4 interaction partially lost following exendin-4 stimulation. Furthermore, the level of GLP-1R–associated NEDD4 was significantly reduced in β-arrestin 2 KD versus control cells, a phenotype that, as for ubiquitination, was already present under vehicle conditions.

DISCUSSION

This study has unveiled a dual effect of β-arrestin 2 in controlling adult β cell GLP-1R function in vivo, with a previously unappreciated positive role during acute signaling that evolves toward a prominent role as a GLP-1R–desensitizing factor during prolonged agonist stimulations. This builds upon our previous in vitro cell line data suggesting GLP-1R signal prolongation following β-arrestin down-regulation (*17*), although these experiments were performed after KD of both β-arrestin isoforms, therefore potentially missing any deleterious effects of imbalanced β-arrestin 1 versus β-arrestin 2 expression that have now come to the fore. To date, there was very limited data on the precise role of this important signaling regulator in the modulation of in vivo GLP-1R responses, despite the known capacity of biased GLP-1RAs with differential G protein over β-arrestin recruitment propensities to modify β cell behaviors (*3*, *5*). Although a previous report had suggested that islets from adult β cell β-arrestin 2–deleted mice have normal ex vivo acute GLP-1R responses, data were obtained from lean male mouse islets (*25*), which do not display significant acute effects in vivo, highlighting the importance of analyzing effects in both sexes and under metabolic stress conditions, which we did here in mice fed a HFHS diet for over 20 weeks, where the β-arrestin 2 effects in both males and females became more apparent.

In the present study, we have identified a more pronounced dependency for β cell β-arrestin 2 to regulate pharmacological GLP-1R responses in females, where we have detected negative acute versus positive prolonged effects of knocking out this signaling regulator in mice fed a chow diet, while, conversely, the in vivo effects observed in chow-fed male mice are more subtle. Note that, while HFHS feeding increases the prominence of both male- and female-specific effects in β cell β-arrestin 2 KO mice, the phenotype of male mice manifests predominantly as improved IPGTT responses 6 hours after exendin-4 administration; while in females, the clearest change relates to defective acute exendin-4 IPGTT responses that subsequently recover with prolonged agonist exposure. While the reasons behind the observed sex differences on the pattern of GLP-1R β-arrestin 2 dependency have not been analyzed here in detail, note that current existing data suggest reduced effectiveness of GLP-1RA treatments for T2D and weight loss in males, while females display instead a higher propensity for adverse side effects to incretin treatment (*45*). Our observations open the door to the possibility that some of the sex differences observed in the clinical responses to incretin therapy are determined by sex dimorphic effects of GLP-1R:β-arrestin 2 interactions.

The identified in vivo phenotype is concomitant with increases in prolonged over acute plasma insulin levels from β-arrestin 2 KO mice and is not present following stimulation with exendin-phe1, an exendin-4–derived GLP-1RA with a single–amino acid substitution biased away from the recruitment of both β-arrestins (*5*), a feature that likely allows it to bypass both the positive and the negative effects of β-arrestin 2 on GLP-1R signaling. We have established the clinical relevance of our observations, as the two currently leading pharmacological GLP-1RAs, namely, semaglutide and tirzepatide (*46*), are both similarly affected by the lack of β cell β-arrestin 2. However, we could not replicate the β cell β-arrestin 2 phenotypes in a whole-animal β-arrestin 2 KO model, suggesting that β-arrestin 2 modulation of GLP-1R action in β cells is compensated by concomitant changes in the response of this or of other GPCRs within other tissues, resulting in a zero net effect in glucose handling. In addition, while the effect of knocking out β cell β-arrestin 2 on physiological islet responses has not been extensively characterized here, we have found no significant effects in oral glucose tolerance under chow diet conditions, but, in agreement with previously published data (*25*), we detected a glucoregulatory defect in HFHS-fed β cell–specific β-arrestin 2 KO mice under vehicle conditions, suggesting a negative impact of β-arrestin 2 deletion on general β cell function, which appears to be accompanied by increases in β cell mass and average islet sizes, as well as enhanced expression of β cell enriched genes, indicating lack of receptor signaling restraint and increased β cell hypertrophy under metabolic stress in the absence of β-arrestin 2.

In addition to the in vivo study, we have also performed a comprehensive ex vivo analysis of GLP-1R downstream signaling responses in β cell β-arrestin 2–deleted islets, revealing an acute cAMP defect by three separate methods, which is no longer present following receptor desensitization after sustained agonist exposure. Moreover, we have determined the involvement of β-arrestin 1 and the cAMP phosphodiesterase PDE4 on this defect, as cAMP production was restored in islets following β-arrestin 1 KD or treatment with the PDE4-specific inhibitor rolipram. These two effects are potentially functionally linked, as β-arrestins can desensitize GPCRs not only by homologous desensitization but also by

recruiting specific PDE isoforms to control the rate of local cAMP degradation (*31*, *47*, *48*), so that abnormally increased GLP-1R:β-arrestin 1 recruitment in the absence of the normally more highly expressed β-arrestin 2 might result in a higher degree of cAMP dampening in adult β cells via augmented recruitment of PDE4, suggesting that alterations in the level of expression of either β-arrestin could lead to defects in β cell GLP-1R signaling. In addition, we observed a reduction in GLP-1R binding to exendin-4–TMR in islets lacking β cell β-arrestin 2: This could again reflect a difference in behavior between both arrestins, as β-arrestin 2 is known to induce higher agonist-binding affinity receptor conformations than β-arrestin 1 for certain GPCRs (*49*, *50*). Other potential mechanisms that might explain the reduction in GLP-1RA binding affinity in the absence of β-arrestin 2 include differences in receptor posttranslational modifications such as the changes in ubiquitination observed during this study, which could have an impact in the capacity of GLP-1Rs to bind to peptide agonists. Overall, our results have unveiled a mechanism by which substitution of β-arrestin 2 by β-arrestin 1 in the absence of the normally β cell predominant isoform is associated with acute defects on GLP-1R signaling, suggesting that the design of biased GLP-1RAs away from recruitment of both β-arrestins should be favored to elicit enhanced sustained GLP-1R responses without triggering acute deficits.

In the present study, we have also shown that reductions in acute GLP-1R–stimulated cAMP generation are propagated toward acute deficits in calcium responses and insulin secretion in islets with β cells that lack β-arrestin 2, an effect that is overturned after sustained exendin-4 stimulations, so that the net effect is an increase in sustained versus acute secretory responses in β cell β-arrestin 2–deleted islets. We have also examined the effects of knocking out β-arrestin 2 in β cells on pan-islet β cell connectivity, a measure of correlation in calcium responses between individual β cells of an islet. We and others have previously shown that β cell connectivity is reduced under glucolipotoxic conditions and can be restored via GLP-1R activation (*38*, *51*). Here, we show that while deletion of β-arrestin 2 from β cells does not affect the acute potentiation of connectivity triggered by GLP-1R agonism in HFHS-fed mice, this increased connectivity is prolonged over time in β cell–specific β-arrestin 2 KO islets, suggesting that β-arrestin 2–dependent GLP-1R desensitization plays an important role in regulating pan-islet interconnectivity responses. In addition, our results also suggest that GLP-1RAs biased away from β-arrestin recruitment might also trigger similar long-term improvements in intra-islet β cell connectivity parameters.

To further our knowledge of the molecular mechanisms underlying the changes in signaling and in vivo glucoregulation observed in the absence of β cell β-arrestin 2, we have assessed GLP-1R trafficking patterns in intact islets, and despite no differences in GLP-1R cell surface levels or internalization in KO islets, we observed a significant reduction in receptor plasma membrane recycling and lysosomal targeting. The lack of effect of β-arrestin 2 in GLP-1R internalization, reminiscent of the behavior of the GIPR in β-arrestin 2 KD adipocytes (*52*), was expected as it has previously been observed using cell lines and has now been validated here in primary islets. On the other hand, the reduced GLP-1R recycling result was not anticipated, as it is opposed to the increased recycling previously observed following reduced recruitment of both β-arrestins to GLP-1Rs stimulated with the G protein–biased compound exendin-phe1 (*5*) and might therefore represent a β-arrestin isoform-specific effect. We nevertheless observed reduced GLP-1R lysosomal targeting, which coincides with the reduced GLP-1R degradation triggered by exendin-phe1 (*5*), as well as receptor redirection to the TGN, a location from where we could now detect enhanced signaling. Overall, our results suggest that absence of β-arrestin 2 results in a unique GLP-1R trafficking signature that is not fully recapitulated by the reduced recruitment of both β-arrestins triggered by biased GLP-1RAs such as exendin-phe1. In addition, using an in vivo technique involving injection of the near-infrared–labeled exendin-4–derivative Ex-4–VT750 into living mice, we could detect a loss of signal from cleared pancreata of control animals at 6 hours over 1 hour after agonist injection that tended to be attenuated in pancreata from β-arrestin 2 KO mice, suggesting retention of pancreatic GLP-1R levels over prolonged agonist stimulation periods in the absence of β-arrestin 2, in agreement with the observed ex vivo reduction in GLP-1R lysosomal targeting and, hence, presumably, GLP-1R degradation.

Most of our observations here are in vivo or ex vivo, but we have also created a rat insulinoma cell model with CRISPR-Cas9–deleted β-arrestin 2 to test our signaling and trafficking hypotheses with further mechanistic detail. Using these cells to investigate compartmentalized signaling, we have confirmed that β-arrestin 2 downregulation triggers reduced GLP-1R signaling efficacy from the plasma membrane without loss of endosomal signaling, and this is accompanied by a reduction in acute (5-min) ERK1/2 and CREB activations in response to exendin-4. We should note, however, that these reductions were measured against a background of increased basal phosphorylation for both signal transduction factors in β-arrestin 2 KD versus control cells. While it is difficult to attribute a specific cause for these differences, as they could be associated with the process of selection of CRISPR-Cas9–targeted cell subpopulations, we cannot exclude that the basal phosphorylation state of cells is specifically increased by the absence of β-arrestin 2.

In addition, in agreement with our islet results, we have observed reduced targeting of active GLP-1Rs to recycling endosomes and lysosomes but increased interaction with the endosome-to-TGN marker Rab9, as well as increased TGN-localized over global cAMP production, suggesting that, as for other GPCRs (*53*), TGN-rerouted GLP-1Rs are able to signal from this intracellular location. Overall, our observations point to a mechanism of signal prolongation in β-arrestin 2–deleted β cells based on the avoidance of receptor degradation and increased access to TGN-based intracellular signaling platforms.

In parallel to studying GLP-1R behaviors, we have also performed a limited number of experiments on GIPR responses in β cell β-arrestin 2 KO mice. Despite similar in vivo tendencies toward reduced sustained over acute glucose levels in KO animals treated with the stable GIP analogue D-Ala2-GIP, these effects did not quite reach statistical significance, suggesting a reduced role of β-arrestin 2 on regulating this receptor compared to the GLP-1R. Accordingly, acute insulin secretion in response to GIP showed only a tendency toward reduction in KO islets from chow-fed animals, although it did reach significance in islets from HFHS-fed mice, with the same tendency toward improvement in prolonged over acute exposures than for the GLP-1R. In addition, in agreement with a reduced influence of β-arrestin 2 on GIPR responses, no differences in plasma membrane or endosomal

signaling were detected in GIP-stimulated β-arrestin 2 KD rat insulinoma cells.

Last, as β-arrestin 2 is known to be required for NEDD4 recruitment and ubiquitination of various GPCRs, including the β_2-adrenergic receptor (*54*), μ-opioid receptor, and V2 vasopressin receptor (*55*), in a mechanism that allows segregation of active receptors toward the degradative pathway following ubiquitin-specific binding to the endosomal sorting complex required for transport (ESCRT) machinery (*56*), we have also investigated whether a similar mechanism would be in place for the GLP-1R. Alterations in this process could potentially explain the observed differences in lysosomal targeting and increased signal prolongation in β-arrestin 2–deleted versus control β cells, particularly as NEDD4 is one of the factors that we have recently identified as a GLP-1R interactor in a mass spectrometry analysis of the receptor β cell interactome. Analysis of ubiquitination has unveiled that the GLP-1R is constitutively ubiquitinated, with exendin-4 stimulation triggering its partial deubiquitination, a pattern also recently observed for the closely related glucagon receptor (*57*). In parallel and closely mimicking these ubiquitination results, NEDD4 was found constitutively recruited to the receptor, with recruitment reduced following exendin-4 exposure. There was a significant reduction in the overall level of GLP-1R ubiquitination and NEDD4 recruitment in cells without β-arrestin 2, which was already present under vehicle conditions. Constitutive GPCR ubiquitination often functions to control receptor trafficking through the biosynthetic pathway (*58*), but we did not find any differences in cell surface GLP-1R levels in control versus β-arrestin 2 KD cells, arguing against this possibility. Ubiquitination of certain GPCRs promotes their basal internalization and lysosomal degradation, whereas deubiquitination often leads to receptor recycling, switching the receptor′s fate and enhancing resensitization (*58*, *59*). Our observations could fit with a role for altered ubiquitination in disrupting the normal GLP-1R intracellular trafficking pattern in the absence of β-arrestin 2.

In conclusion, we present here a comprehensive assessment of the effects of knocking out β-arrestin 2 from primary β cells on pharmacological GLP-1R responses, demonstrating reduced acute but enhanced long-term in vivo glucoregulation that manifests differently in males versus females. These effects correlate with a β-arrestin 1– and PDE4-dependent defect in cAMP production and alterations in GLP-1R trafficking, consisting of the diversion of active GLP-1Rs from the lysosome to the TGN, as well as an overall reduction in GLP-1R ubiquitination and NEDD4 recruitment. Our study analyzes in detail the consequences of selective β-arrestin 2 deletion from adult primary β cells on GLP-1R behaviors. These data have important implications for the rational design of future GLP-1RAs with increased therapeutic windows and could lead to very prominent cumulative effects in the context of repeated administration of GLP-1RAs in chronic regimes such as those required in T2D and obesity treatments.

Our study has several limitations, including difficulties in adjusting experimental conditions to translate in vivo acute and prolonged observations to the corresponding ex vivo trafficking and signaling assessments. Moreover, data for in vivo responses to semaglutide and tirzepatide were collected from a mixed sex cohort of HFHS-fed mice and would have been more informative if collected from separate male and female mouse cohorts. In addition, our trafficking studies are performed with fluorescently labeled exendin-4 and exendin-9 as a proxy for endogenous GLP-1R localization, which is not entirely accurate. Better experiments could be performed in the future using animals genetically modified to express SNAP- or HALO-tagged GLP-1Rs from the endogenous gene locus. Last, mechanistic studies in INS-1 832/3 cells will require validation in primary islets and experiments should be designed to establish causation from correlative data such as the effect of reduced GLP-1R ubiquitination in modulating GLP-1R postendocytic trafficking and signaling.

MATERIALS AND METHODS

Animal studies

All in vivo procedures were approved by the U.K. Home Office under the Animals (Scientific Procedures) Act 1986 (Project Licence number PA03F7F0F, P0A6474AE, and PP7151519 to I. Leclerc, B. Owen, and A. Martinez-Sanchez, respectively) and from the local ethical committee (Animal Welfare and Ethics Review Board) at the Central Biological Services unit of Imperial College London. Animals were housed in groups of up to five adult mice in individually ventilated cages under controlled conditions (21° to 23°C; 12-hour light:12-hour dark cycles). Ad libitum access to standard chow diet was provided. For HFHS diet studies, animals were put on a 58 kcal % fat and sucrose diet (D12331, Research Diet, New Brunswick, NJ) ad libitum for the indicated time periods.

Genomic DNA extraction and genotyping

Genotyping was carried out using ear samples collected from weaned animals. Extraction of genomic DNA was performed in an alkaline lysis buffer [25 mM NaOH and 0.2 mM EDTA (pH 8.0) in distilled H_2O] for 1 hour at 95°C and subsequently neutralized by the addition of 13 mM tris-HCl (pH 7.4). The genomic DNA was used as a template for a PCR with the appropriate primers (sequences provided in Table 1) and the Phire Green Hot Start II DNA polymerase (F124-L, Thermo Fisher Scientific, MA, USA). The PCR products were visualized on 1 to 2% agarose gels using a Bio-Rad ChemiDoc imaging system.

Generation of β cell–specific β-arrestin 2 KO and control mice

Transgenic mouse models were generated on a C57BL/6 background using the Cre/Lox system. The Pdx1-Cre-ERT (Cre recombinase under the control of the *Pdx1* promoter conjugated to a mutant estrogen receptor sequence) mice were bred in-house, whereas the floxed β-arrestin 2 (Barr2) mice, in which exon 2 is flanked by loxP sites ($Barr2^{fl/fl}$), were provided by M. Caron (Duke University, USA). Pdx1-Cre-ERT mice were crossed with $Barr2^{fl/fl}$ mice in subsequent breeding pairs until mice hemizygous for the Pdx1-Cre-ERT and homozygous for floxed Barr2 ($Barr2^{fl/fl}$) were obtained. These mice were then bred to produce $Barr2^{fl/fl}$ mice with or without Pdx1-Cre-ERT. $Barr2^{fl/fl}$ mice injected with tamoxifen were used as a control, since tamoxifen administration is a potential confounder (*60*, *61*). Tamoxifen-treated Pdx1-Cre-ERT mice do not have altered β cell function compared with WT littermates (*62*); thus, they were not used as an additional control. Tamoxifen (T5648, Sigma-Aldrich) dissolved at 20 mg/ml in corn oil (C8267, Sigma-Aldrich) was injected at a dose of 100 mg/kg intraperitoneally at 8 weeks of age for five consecutive days to induce Cre recombination, and, thus, β cell–specific Barr2 KO mice and littermate

Table 1. Primers used for genotyping PCR and qPCR.

Gene	Species	Forward sequence (5′ → 3′)	Reverse sequence (5′ → 3′)	Application
Barr2 flox	Mouse	GAGTCACTGTATGGGTCCCTG	TTGCTGTTCGATGCTACATAACTC	Genotyping
Barr2	Mouse	GCGCGACTTTGTAGATCACC	AAGACAGGCCCAGTACATCC	qPCR
Barr2	Rat	AAGTCGAGCCCTAACTGCAA	GCCCAGTACATCCAGGTCTT	qPCR
Barr1	Rat	TAACGTGCAGTCCTTCCCAC	GAAGGTTTGGCGGGATCTCA	qPCR
Barr1	Mouse	CCAGACAGTTCCTTATGTCAGACAA	TTCTCCGTGGTAATAGATCTCCTTATC	qPCR
β-Actin	Mouse and rat	CACTGTCGAGTCGCGTCC	TCATCCATGGCGAACTGGTG	qPCR
Pdx1	Mouse	CCAAAGCTCACGCGTGGA	TGTTTTCCTCGGGTTCCG	qPCR
CAMPER mut (flox)	Mouse	GGTCAGCTTGCCGTAGGTG	GTCCAAGCTGAGCAAAGACC	Genotyping
CAMPER wt (no flox)	Mouse	CAGGACAACGCCCACACA	AAGGGAGCTGCAGTGGAGTA	Genotyping
CreERT	Mouse	AGCGATGGATTTCCGTCTCT	CACCAGCTTGCATGATCTCC	Genotyping
GCaMP6f mut (flox)	Mouse	ACGAGTCGGATCTCCCTTTG	CCGAAAATCTGTGGGAAGTC	Genotyping
GCaMP6f wt (no flox)	Mouse	AAGGGAGCTGCAGTGGAGTA	CCGAAAATCTGTGGGAAGTC	Genotyping
Ins1	Mouse	GCTGGTGGGCATCCAGTAA	AATGACCTGCTTGCTGATGGT	qPCR
Ins2	Mouse	TGGCTTCTTCTACACACCCATGTCCC	ACTGATCTACAATGCCACGCTTCTGCT	qPCR
MafA	Mouse	CTTCAGCAAGGAGGAGGTCATC	CGTAGCCGCGGTTCTTGA	qPCR
Kcjn11	Mouse	CACGGCGGGATAAGTCTACCT	AATCATTTGCCCCCTTCTTGT	qPCR
Acot7	Mouse	AGATGATTGAGGAGGCCGG	ACAGCGCTCCCCATTCTG	qPCR
Ldha	Mouse	ATGAAGGACTTGGCGGATGA	ATCTCGCCCTTGAGTTTGTCTT	qPCR
Slc16a1	Mouse	GCTTGGTGACCATTGTGGAAT	CCCAGTACGTGTATTTGTAGTCTCCAT	qPCR

controls were generated. Experiments were conducted at least 10 days after the last tamoxifen injection to allow for a sufficient tamoxifen washout period.

R26-Cre-ERT2 mice were provided by T. Rodriguez, Imperial College London, UK. This mouse line allows for ubiquitous tamoxifen-inducible Cre recombination, as a Cre-ERT2 cassette is inserted into the Rosa26 locus. These mice were crossed with Barr2$^{fl/fl}$ mice until the generation of Barr2$^{fl/fl}$ mice with or without expression of R26-Cre-ERT2. Tamoxifen administration was performed as described above to produce inducible whole-body Barr2 KO and littermate control mice.

Mice that express the genetically encoded calcium indicator GCaMP6f Cre-dependently were used for in vivo calcium imaging experiments using islets implanted into the anterior chamber of the eye (*37*). GCaMP6f mice were bred in-house and crossed with Pdx1-Cre-ERT Barr2$^{fl/fl}$ mice to generate GCaMP6f-Pdx1-Cre-ERT Barr2$^{fl/+}$ breeders. Subsequently, GCaMP6f-Pdx1-Cre-ERT Barr2$^{fl/fl}$ and GCaMP6f-Pdx1-Cre-ERT Barr2$^{+/+}$ mice were obtained from the crossings and injected with tamoxifen to yield GCaMP6f^{+} β cell Barr2 KO and control GCaMP6f^{+} Barr2 WT mice, respectively. In this case, the presence of the Cre recombinase is essential for GCaMP6f expression, as the conditional allele contains a loxP-flanked cassette.

Similarly, conditional expression of the biosensor ^{T}EPAC^{VV} was used for imaging of cAMP dynamics: *CAMPER* reporter mice (*29*) were purchased from the Jackson Laboratory (stock no. 032205) and crossed with Pdx1-Cre-ERT Barr2$^{fl/fl}$ mice to generate *CAMPER*-Pdx1-Cre-ERT Barr2$^{fl/+}$ breeders. *CAMPER*-Pdx1-Cre-ERT Barr2$^{fl/fl}$ and *CAMPER*-Pdx1-Cre-ERT Barr2$^{+/+}$ mice were obtained from the crossings and injected with tamoxifen resulting in *CAMPER*$^{+}$ β cell–specific Barr2 KO and control *CAMPER*$^{+}$ Barr2 WT mice, respectively.

Peptides

Peptides were produced by Wuxi AppTec Co. Ltd., Shanghai, China, using standard solid-phase peptide synthesis. Mass spectrometric confirmation of peptide identity and high-performance liquid chromatographic purity assessment were provided by the manufacturer (confirmed >90% purity). TMR-labeled exendin-4 (exendin-4–TMR) and exendin-9 (exendin-9–TMR) have been described and validated before (*63*). VivoTag-750–conjugated exendin-4 (exendin-4–VT750), used for OPT imaging, was synthesized and provided by S. Goudreau (ImmuPharma Group, Pessac, France).

Intraperitoneal glucose tolerance tests

IPGTTs using glucose (2 g/kg) were performed immediately and at the indicated time points after agonist injection (coadministered with glucose in the acute test) to examine acute and prolonged glycemic responses to GLP-1R agonism. The animals were fasted for 2 hours before the tests starting at 8 a.m., the morning of the experiment. Blood samples were analyzed at 0, 10, 30, and 60 min after glucose ± agonist injection using a Contour glucometer (Bayer) and strips. The food was topped up at the end of the 6-hour IPGTT for agonists with shorter half-life (exendin-4, exendin-phe1, and D-Ala2-GIP) or at the end of each IPGTT for agonists with longer half-life (tirzepatide and semaglutide).

Additional in vivo metabolic tests

For oral glucose tolerance tests (OGTTs) and IPITTs, mice were fasted 5 hours before experiments. For OGTTs, glucose (2 g/kg of body weight) was administered directly into the gut via oral gavage and blood glucose levels determined by tail venepuncture using an automatic glucometer (Accu-Chek, Roche) at 0, 10, 30, and 60 min after the glucose load. For IPITTs, mice received an intraperitoneal injection of insulin (0.75 IU/kg), and blood glucose levels were determined as above at 0, 15, 30, and 60 min after insulin injection.

Measurement of in vivo plasma insulin levels

During selected IPGTTs, blood samples were collected at the indicated times for plasma insulin analysis. Whole blood samples were collected in potassium EDTA cuvettes (Microvette CB 300, 16.444.100, Sarstedt) and centrifuged at 500*g* for 10 min at 4°C. The supernatant (plasma) was collected in fresh Eppendorf tubes and kept at −80°C until analysis with a mouse insulin enzyme-linked immunosorbent assay (ELISA) kit (ultrasensitive mouse insulin ELISA kit, 90080, Crystal Chem), performed according to the manufacturer's instructions with two technical replicates per sample.

α And β cell mass quantification

Pancreata from β cell β-arrestin 2 KO and control littermates were dissected and fixed in 4% paraformaldehyde (PFA) for 24 hours. The tissues were washed twice in phosphate-buffered saline (PBS) and left in 70% ethanol until wax embedding. For each sample, three 5-μm-thick tissue sections separated by 300 μm were stained with guinea pig anti-insulin antibody (undiluted, IR002, Dako; Alexa Fluor 488 secondary antibody, Invitrogen) and mouse antiglucagon antibody (1:500; G2654, Sigma-Aldrich; Alexa Fluor 568 secondary antibody, Invitrogen). Images were captured using a widefield Zeiss Axio Observer inverted microscope. Glucagon- and insulin-positive areas were determined as previously described (*64*) and expressed relative to the total pancreas area imaged. The average islet size was approximated by adding the surface of β cells and α cells for each islet per section, calculating the average, and producing the overall average for three separate sections per sample. ImageJ v1.53c was used for image analysis.

Isolation and culture of pancreatic islets

For ex vivo islet experiments, pancreatic islets were isolated from appropriate KO and littermate control mice. Pancreata were infused via the common bile duct with RPMI 1640 medium (R8758, Sigma-Aldrich) containing collagenase (1 mg/ml) from *Clostridium histolyticum* (S1745602, Nordmark Biochemicals), dissected, and incubated in a water bath at 37°C for 10 min. Islets were subsequently washed and purified using a Histopaque gradient (Histopaque-1119, 11191, Sigma-Aldrich; and Histopaque-1083, 10831, Sigma-Aldrich). Isolated islets were allowed to recover overnight at 37°C in 5% CO_2 in RPMI 1640 supplemented with 10% (v/v) fetal bovine serum (FBS) (F7524, Sigma-Aldrich) and 1% (v/v) penicillin/streptomycin (P/S) solution (15070-063, Invitrogen).

Implantation of islets into the anterior eye chamber

For chow diet calcium in vivo imaging, two 8-week-old female littermates with WT and KO genotypes for Barr2: one control (GCaMP6f heterozygous, Pdx1-Cre-ERT$^+$ Barr2$^{+/+}$) and one KO (GCaMP6f heterozygous, Pdx1-Cre-ERT$^+$ Barr2$^{fl/fl}$) were injected with tamoxifen for five consecutive days to induce GCaMP6f and Barr2 flox recombination. At 12 weeks of age, the animals were euthanized, and pancreatic islets were isolated. The day after the islet isolation, these were transplanted into the anterior chamber of the eye of six 12-week-old male WT C57BL/6 J acceptors as in (*37*). Three of the acceptors received control and three KO donor islets. The success of the implantation was verified after 4 weeks, and imaging experiments were carried out 8 weeks after the operation.

For calcium imaging under HFHS diet conditions, two 8-week-old female littermates with WT and KO genotypes for β-arrestin 2 were concomitantly injected with tamoxifen and introduced to HFHS diet. Diet administration was continued for 8 weeks before animal euthanasia and islet isolation. The islets were implanted in the eyes of three 12-week-old female acceptors that had been on HFHS diet for 4 weeks. The acceptors received KO islets in their left eye and control islets in their right eye. The HFHS diet was continued throughout the study. Imaging experiments were carried out 4 to 5 weeks after the operation.

In vivo calcium imaging

On the days of the imaging, the acceptor mice for chow diet experiments were injected intraperitoneally with either vehicle (saline) or exendin-4 (10 nmol/kg). For HFHS diet experiments, the mice were injected with glucose (2 g/kg) with or without exendin-4 (10 nmol/kg). All acceptor mice received both treatments using a cross-over study design with block randomization. Images were captured 30 min and 5 or 6 hours after injection using a Nikon Eclipse Ti microscope with an ORCA-Flash 4.0 camera (Hamamatsu) and Metamorph software (Molecular Devices).

During the imaging experiments, general anesthesia was induced using isoflurane, and time-lapse confocal microscopy performed using the 488-nm excitation channel for 181 frames with 800-ms exposure per frame. During the chow diet calcium imaging, mice injected with exendin-4 received intraperitoneal injections of glucose (2 g/kg) [20% (w/v) glucose solution] to increase blood glucose levels shortly before image acquisition.

Wave index assignment

Pancreatic β cells are coupled such that healthy pulsatile insulin secretion is associated with pan-islet calcium oscillations or waves. We defined a wave index to objectively measure the proportion of an islet cross-section involved in calcium oscillatory activity. The wave index of each implanted islet was determined using Fiji: Whole-islet mean intensity readouts were obtained using a manual motion-correction macro developed by S. Rothery [National Heart and Lung Institute (NHLI) Facility for Imaging by Light Microscopy (FILM), Imperial College London]. Next, we determined the average whole-islet calcium readout in an image sequence and multiplied this number by 1.2 to determine genuine calcium activity in islets. Using this value, the image sequences were thresholded to select cross-sectional areas with genuine calcium wave activity, expressed as a percentage relative to the whole-islet cross-sectional area imaged. The highest percentage value in each image sequence was used to determine the islet's wave index for that imaging session. Values were then corrected for the fact that, in implanted islets, approximately 20% of the imaged islet cross-section is covered by blood vessels. Using this method, we were able to determine the presence of four types of calcium wave activity, in line with earlier studies (*38*): Islets where

we observed activity over 0 to 25% of corrected area were characterized as type 1; if 26 to 50% of the islet cross-section was active, these were typical of type 2; if 51 to 75% of the cross-section was active, islets were classified as displaying type 3 activity; and, last, type 4 activity was assigned to islets where 76 to 100% of the cross-section was active.

Waveform analysis

The wavelength, FWHM, and amplitude were determined for the calcium traces of all islets imaged using MATLAB. Briefly, GCaMP6f fluorescence intensity traces were normalized to Fmin, and a cutoff value of 1.2 for genuine calcium activity was determined. Peaks in the data were determined using the derivative of the waveform for a given minimum peak height and distance between peaks. Frequency and wavelength were then calculated on the basis of peak location and value. Amplitude and FWHM were calculated as an average of all individual peak amplitude and FWHM for a given dataset. Data without distinct peaking behavior were discarded as noise.

Connectivity analysis

For single-cell connectivity analysis, individual β cells were identified visually using the negative shadow of nuclei as a guidance for region of interest (ROI) placement. ROIs were subcellular and their *XY* coordinates, and changes in mean intensity were measured. Using a MATLAB script, Pearson R correlation analysis of calcium time traces was performed. Calcium traces were normalized and smoothed using prospective time points in the dataset. Pearson correlation between individual cell pairs was determined, excluding autocorrelation. An R value of 0.25 was set as the threshold to signify a connection. Data were resampled using boot strapping to increase accuracy of findings (R values with $P < 0.001$ were deemed statistically significant). R values were binned as follows: 0.25 to 0.50, 0.5 to 0.75, and 0.75 to 1, and considered to signify weak, medium, or strong connections, respectively. Cartesian line maps showing β cell connectivity were generated on the basis of cell *XY* coordinates, and connections were assigned yellow, green, or red depending on strength of connections. Heatmap matrices show the R values for each individual cell pair, with an R value of 1 assigned for autocorrelation.

Ex vivo calcium imaging

Imaging of whole-islet Ca^{2+} dynamics was performed 24 hours after isolation, essentially as previously described (*51*, *64*). Islets from individual animals were preincubated for 1 hour in Krebs-Ringer bicarbonate–Hepes (KRBH) buffer (140 mM NaCl, 3.6 mM KCl, 1.5 mM $CaCl_2$, 0.5 mM $MgSO_4$, 0.5 mM NaH_2PO_4, 2 mM $NaHCO_3$, 10 mM Hepes, saturated with 95% O_2/5% CO_2; pH 7.4) containing 0.1% (w/v) bovine serum albumin (BSA), 6 mM glucose (KRBH G6), and the Ca^{2+}-responsive dye Cal-520 AM (AAT Bioquest). For chow diet experiments, islets were excited at 488 nm and images captured at 0.5 Hz using a Zeiss Axiovert microscope equipped with a 10×/0.5 numerical aperture objective and a Hamamatsu image–EM camera coupled to a Nipkow spinning-disk head (CSU-10, Yokogawa). Volocity software (PerkinElmer) provided a visualization interface, while islets were maintained at 37°C on a heated stage constantly perifused with KRBH buffer containing G6 ± 100 nM exendin-4, 11 mM glucose, or 20 mM KCl. For HFHS diet experiments, islets were imaged with a Zeiss LSM-780 inverted confocal laser-scanning microscope in a 10× objective from the FILM Facility at Imperial College London. Treatments were manually added to the islet dishes by pipetting at the indicated time points. To ensure that the islets remained stable, these were pre-encased into Matrigel (356231, Corning) and imaged on glass-bottom dishes (P35G-1.5-10-C, MatTek). Raw fluorescence intensity traces from whole-islet ROIs were extracted using ImageJ v1.53c. Responses were plotted relative to the average fluorescence intensity per islet during the 6 mM glucose baseline period, before agonist addition.

Islet GLP-1R trafficking experiments

Ex vivo islet GLP-1R trafficking experiments were carried out using intact islets treated in full medium in 24-well suspension plates for the indicated time points. The islets were loaded at the center of a glass-bottom dish in RPMI 1640 without phenol red (32404014, Thermo Fisher Scientific). *Z*-stacks were obtained by confocal microscopy in a NHLI FILM Zeiss LSM-780 inverted confocal laser-scanning microscope and a 20× objective. The images were analyzed using ImageJ v1.53c and processed using Z project with maximum intensity projections, and the mean intensity of selected ROIs was measured.

Ex vivo islet cAMP imaging

CAMPER islet FRET imaging

Intact islets from *CAMPER*-expressing mice (above) were used 24 to 48 hours following isolation. Ten to 20 islets from individual mice were encased into Matrigel on glass-bottom dishes and imaged for FRET between cyan fluorescent protein (CFP) (donor) and yellow fluorescent protein (YFP) (acceptor) with CFP excitation and both CFP and YFP emission settings in an NHLI FILM Zeiss LSM-780 inverted confocal laser-scanning microscope and a 20× objective to capture time-lapse recordings with image acquisition every 6 s. The treatments were manually added by pipetting. Specifically, for acute cAMP studies, islets were imaged in KRBH buffer containing 0.1% (w/v) BSA and 6 mM glucose (KRBH G6) for 1 min, and then exendin-4 at 100 nM was added and imaged for 6 min before addition of isobutyl methylxanthine (IBMX) at 100 μM for the final 2 min of the acquisition. For acute rolipram experiments, islets were captured in KRBH G6 for 1 min before addition of 100 nM exendin-4, 10 μM rolipram, or a combination of the two for 5 min. Last, for overnight experiments, the islets were incubated in 24-well suspension plates in full medium with 1 nM exendin-4 for 16 hours overnight, then washed for 30 min in KRBH G6, and imaged for 2 min in this buffer; then 1 nM GLP-1 was added; and, last, after 4 min, 100 μM IBMX was added and imaged for the final 2 min of the acquisition. Raw intensity traces for YFP and CFP fluorescence were extracted from whole-islet ROIs using ImageJ v1.53c and CFP/YFP ratios calculated for each ROI and time point. Responses were plotted relative to the average fluorescence intensity per islet during the 6 mM glucose baseline period, before agonist addition.

Islet cAMP imaging with cADDis

Islets were infected with cADDis (Green Gi cADDis cAMP Assay Kit, Montana Molecular), a genetically encoded biosensor for cAMP packaged in a BacMam viral vector, following the manufacturer's instructions, 24 hours after isolation. Infected islets were then imaged 24 hours after infection. Islets were encased into Matrigel on glass-bottom dishes and imaged at 488 nm in an NHLI

FILM Zeiss LSM-780 inverted confocal laser-scanning microscope and a 10× objective for time-lapse recordings with image acquisitions every 6 s. Treatments were manually added by pipetting. For acute cAMP studies, islets were imaged as above in KRBH buffer containing 0.1% (w/v) BSA and 6 mM glucose (KRBH G6) for 2 min to record the baseline, then exendin-4 at 100 nM was added, and islets were imaged for 5 min before addition of a mixture of 100 μM IBMX and 10 μM forskolin for the final 2 min of the acquisition. For overnight experiments, the islets were incubated in 24-well suspension plates in full medium with 1 nM exendin-4 for 16 hours overnight, then washed for 30 min in KRBH G6, and imaged for 2 min in this buffer; then 1 nM GLP-1 was added; islets were imaged for 5 min; and, last, a mixture of 100 μM IBMX and 10 μM forskolin was added and imaged for the final 2 min of the acquisition. Raw intensity traces for GFP fluorescence were extracted from whole-islet ROIs using ImageJ v1.53c, and mean intensities were calculated for each ROI and time point. Responses were plotted relative to the average fluorescence intensity per islet during the 6 mM glucose baseline period, before agonist addition.

β-Arrestin 1 siRNA KD

For β-arrestin 1 (Barr1) siRNA studies, *CAMPER* primary mouse islets were dispersed by trituration in 0.05% trypsin/EDTA for 3 min at 37°C and seeded at the center of poly-D-lysine–coated glass-bottom dishes. Dispersed islets were allowed to recover overnight before treatment with Accell mouse Arrb1 (109689) siRNA-SMARTpool (E-040976-00-0005, Horizon Discovery) or nontargeting control siRNA (D-001910-01-05, Horizon Discovery) according to the manufacturer's instructions using the recommended siRNA buffer (B-002000-UB-100, Horizon Discovery) and serum-free delivery medium (B-005000-100, Horizon Discovery). Studies took place 72 hours after siRNA treatment addition.

Islet immunostaining for confocal co-localization

For immunostaining and fluorescence confocal microscopy, exendin-4–TMR–treated islets were fixed using 4% PFA and stored in PBS. After a 10-min permeabilization with PBS containing 0.5% (v/v) Triton X-100, the islets were washed once with PBS and incubated in blocking buffer [PBS, 0.1% (v/v) Tween 20, 1% (w/v) goat serum, and 1% BSA] for 30 min. Primary antibody against Lamp1 (1D4B, Developmental Studies Hybridoma Bank) or TGN38 (2F7.1, MA3-063, Thermo Fisher Scientific) in blocking buffer was added overnight, while secondary anti-rat or anti-mouse Alexa Fluor 488 (Thermo Fisher Scientific) was incubated for 30 min at room temperature. Islets were loaded onto glass-bottom dishes in PBS and *Z*-stacks acquired by confocal microscopy with an Imperial College London NHLI FILM Zeiss LSM-780 inverted confocal laser-scanning microscope and a 63×/1.4 numerical aperture oil immersion objective for Lamp1 and an Imperial College London NHLI FILM Leica Stellaris 8 inverted confocal microscope and a 63×/1.4 numerical aperture oil immersion objective for TGN38. Images were analyzed as maximum intensity projections using ImageJ v1.53c, with the Coloc 2 plugin used for colocalization analysis.

Optical projection tomography

Whole-body (R26-Cre-ERT2) β-arrestin 2 KO and control mice were injected intraperitoneally with exendin-4–VT750 (100 nmol/kg). After 1 hour or 6 hours, the mice were euthanized using an overdose of anesthetic (Euthatal solution for injection, Merial) and transcardially perfused with PBS, followed by 4% PFA for perfusion fixation. The pancreas and brain were dissected, washed once in PBS, and placed in 4% PFA for 1 to 6 hours. The tissues were subsequently optically cleared using the 3DISCO protocol (*65*). Dehydration was achieved by incubating in increasing concentrations (1× 50%, 1× 70%, 1× 80%, and 3× 100%) of tetrahydrofuran (401757, Sigma-Aldrich) for 10 to 16 hours each time. Benzyl ether (108014, Sigma-Aldrich) was subsequently added for 16 hours.

The samples were imaged using an OPT microscope built by J. McGinty and P. French, Imperial College London. Scripts developed in MATLAB (MathWorks) by J. McGinty were used for image acquisition, reconstruction, global scaling, and region segmentation. Quantification of object volumes and mean intensity was performed using 3D Objects Counter in ImageJ v1.53c. 3D images were visualized using Volocity software (Quorum Technologies Inc.).

Cell culture

Cell lines were cultured in humidified incubators at 37°C in 5% CO_2. Male insulinoma INS-1 832/3 cells (*66*) (a gift from C. Newgard, Duke University, USA) were grown in RPMI 1640 supplemented with 10% FBS, 1% P/S, 10 mM Hepes (H0887, Sigma-Aldrich), 1 mM sodium pyruvate (11360070, Thermo Fisher Scientific), and 0.05 mM 2-mercaptoethanol (M3148, Sigma-Aldrich). HEK293T cells were maintained in Dulbecco's modified Eagle's medium glucose (4500 mg/liter; D6546, Sigma-Aldrich) with 10% FBS and 1% P/S. Cell lines were screened routinely for mycoplasma contamination.

Generation of lentiviral CRISPR vectors and transduction of INS-1 832/3 cells

For KD of β-arrestin 2 in INS-1 832/3 cells, a lentiviral CRISPR approach was used. Two guide RNA (gRNA) sequences were cloned using a pScaffold-H1 (118152, Addgene) on a lentiCRISPR v2 backbone (52961, Addgene), using a protocol adapted from (*67*). The gRNA sequences were 5′-GAAGTCGAGCCCTAACTGCA-3′ and 5′-ACCGGTATTTGAAGCCTCTT-3′ (reverse complement). The backbone vector was predigested with FastDigest Esp3I (FD0454, Thermo Fisher Scientific) for 2 hours at 37°C and purified using Monarch DNA Gel Extraction Kit (T1020S, New England Biolabs) before digestion ligation with the gRNA-pScaffold-H1 using FastDigest Esp3I and T7 Ligase (M0318L, New England Biolabs).

To produce lentiviral particles, HEK293T cells were cotransfected with the gRNA lentiviral vector (or the vector without gRNAs—control empty vector), as well as the packaging (psPAX2) and envelope (pMD2.G) vectors using a calcium phosphate protocol. Viral supernatants were harvested 48 and 72 hours after transfection, filtered using a 0.45-μm Millex-HV filter, and concentrated by 20% sucrose gradient ultracentrifugation in an Optima XPN-100 ultracentrifuge at 26,000 rpm at 4°C for 2 hours in a SW32 Ti swinging bucket rotor (Beckman Coulter). Viral particles were resuspended in PBS and stored at −80°C.

INS-1 832/3 cells were transduced with appropriate amounts of lentiviruses, followed by addition of puromycin (4 μg/ml) 72 hours after transduction. Transduction was performed with the control empty vector (INS-1 832/3 EV) or the vector with gRNAs 1 and 2 (INS-1 832/3 g1-2) to generate β-arrestin 2 KDs. The puromycin

was replaced every 2 to 3 days for a total of 2 weeks to induce the selection of transduced cells. The surviving cells were subsequently cultured in full medium in the absence of puromycin and tested for *ARRB2* gene expression using qPCR.

Ex vivo insulin secretion assays

Isolated islets used for insulin secretion assays were treated in 24-well nonadherent plates. Ten islets were used per well with three technical replicates per condition. For acute studies, islets were preincubated for 1 hour in KRBH buffer (described in in vitro calcium imaging) containing 1% (w/v) BSA (10775835001, Roche) and 3 mM glucose before incubation with 11 mM glucose ± agonists in KRBH in a shaking 37°C water bath (80 rpm) for 1 hour. For overnight studies, preincubation was carried out in RPMI 1640 medium containing FBS, P/S, and 3 mM glucose, followed by treatment with medium containing 11 mM glucose ± agonists for 16 hours. At the treatment end, supernatants containing the secreted insulin were collected, centrifuged at 1000*g* for 5 min, and transferred to fresh tubes. To determine total insulin contents, islets were lysed using acidic ethanol [75% (v/v) ethanol and 1.5 mM HCl]. The lysates were sonicated 3 × 10 s in a water bath and centrifuged at 10,000*g* for 10 min, and the supernatants were collected. The samples were stored at −20°C until the insulin concentration was determined using an Insulin Ultra-Sensitive HTRF Assay kit (62IN2PEG, Cisbio, Codolet, France) according to the manufacturer's instructions. GraphPad Prism 9.0 was used for the generation of the standard curve and sample concentration extrapolation. The total insulin content was calculated by adding the secreted insulin to the insulin content of the lysates.

HTRF cAMP assays

Intact primary mouse islets were dispersed by trituration in 0.05% trypsin/EDTA for 3 min at 37°C. After stimulation, dispersed islets were lysed and cAMP assayed by HTRF immunoassay (cAMP Dynamic 2, 62AM4PEB, Cisbio, Codolet, France).

Transfection of plasmid DNA

Transient transfection of cell lines with plasmid DNA was achieved with Lipofectamine 2000 Transfection Reagent (11668027, Thermo Fisher Scientific) according to the manufacturer's instructions. Briefly, appropriate amounts of DNA were incubated for 5 min in Opti-MEM reduced serum medium (31985070, Thermo Fisher Scientific) and gently mixed with an equal volume of Lipofectamine 2000 in Opti-MEM. After 20 min, the mix was added dropwise to cells in culture medium without P/S and incubated for 4 to 6 hours. The medium was then removed, and full culture medium was added. Assays were performed 24 or 48 hours after transfection depending on the experiment.

RNA extraction and qPCR

Samples were lysed in TRIzol reagent (15596-018, Invitrogen) and briefly vortexed for homogenization. Phase separation was achieved by chloroform (C2432, Sigma-Aldrich) addition, and the upper aqueous phase was collected. RNA was recovered by overnight precipitation with isopropanol (11388461, Thermo Fisher Scientific). Following cDNA synthesis using MultiScribe Reverse Transcriptase (4311235, Thermo Fisher Scientific) according to the manufacturer's instructions, qPCR was performed using SYBR Green Technology (Fast SYBR Green Master Mix, 4385616, Invitrogen) on an Applied Biosystems 7500 real-time PCR system. Data were analyzed using the $2^{-\Delta\Delta Ct}$ method (*68*). A list of primer sequences is provided in Table 1.

cAMP FRET imaging using global and TGN-targeted $^{T}EPAC^{VV}$

The global $^{T}EPAC^{VV}$ construct was provided by K. Jalink, the Netherlands Cancer Institute, Netherlands (*69*). The TGN-targeted version was made in-house by in-frame cloning of the golgin-97, RanBP2α, Imh1p and p230/golgin-245 (GRIP) domain of GolginA1 (pCMV6-KL5-GolginA1, Origene) at the C-terminal end of $^{T}EPAC^{VV}$. INS-1 832/3 EV and g1-2 cells were plated in 48 wells and transfected with 0.5 μg of plasmid DNA. After 24 hours, cells were trypsinized and seeded onto glass-bottom dishes. Forty-eight hours after transfection, cells were imaged in RPMI 1640 without phenol red (32404014, Thermo Fisher Scientific) with an NHLI FILM Zeiss LSM-780 inverted confocal laser-scanning microscope using a 20× objective to capture a time-lapse recording of CFP/YFP FRET as described for *CAMPER* islets with image acquisition every 6 s and treatments manually added by pipetting. Cells were imaged for 1 min to record a baseline, and then exendin-4 at 100 nM was added and imaged for 4 min before addition of 100 μM IBMX and 10 μM forskolin for the final 2 min of the acquisition. Raw intensity traces for YFP and CFP fluorescences were extracted from individual cell ROIs using ImageJ v1.53c, and CFP/YFP ratios were calculated for each ROI and time point. Responses were plotted relative to the average fluorescence intensity per islet during the 6 mM glucose baseline period, before agonist addition.

NanoBiT complementation and NanoBRET assays

For Nb37-based bystander complementation assays, the plasmids used were a gift from A. Inoue, Tohoku University, Japan (*40*). Nb37 cDNA (synthesized by GenScript with codon optimization) was C-terminally fused to SmBiT with a 15–amino acid flexible linker (GGSGGGGSGGSSSGGG), and the resulting construct was referred to as Nb37-SmBiT. The C-terminal KRAS CAAX motif (SSSGGGKKKKKKSKTKCVIM) was N-terminally fused with LgBiT (LgBiT-CAAX). The Endofin FYVE domain (amino acid region Gln^{739}-Lys^{806}) was C-terminally fused with LgBiT (Endofin-LgBiT). $G\alpha_s$ (human, short isoform), $G\beta_1$ (human), $G\gamma_2$ (human), and RIC8B (human, isoform 2) plasmids were inserted into pcDNA 3.1 or pCAGGS expression vectors. INS-1 832/3 EV or g1-2 cells were seeded in six-well plates and cotransfected with 0.2 μg of SNAP-GLP-1R or SNAP-GIPR; 0.5 μg of $G\alpha_s$, $G\beta_1$, and $G\gamma_2$; 0.1 μg of RIC8B; 0.1 μg of CAAX-LgBiT; or 0.5 μg of Endofin-LgBiT with 0.1 or 0.5 μg of Nb37-SmBiT (1:1 ratio), respectively.

For NanoBRET localization assays, cells were seeded in 12-well plates and cotransfected with SNAP–GLP-1R–NLuc (generated in-house) and KRAS-, Rab4-, Rab5-, Rab9-, or Rab11-Venus (gifts from N. Lambert, Augusta University, USA, and K. Pfleger, The University of Western Australia, Australia). For KRAS-, Rab5-, and Rab11-Venus, 0.5 μg were cotransfected with 0.5 μg of SNAP–GLP-1R–NLuc, while for Rab4- and Rab9-Venus, 0.25 μg were cotransfected with 0.1 μg of SNAP–GLP-1R–NLuc.

Twenty-four hours after transfection, cells were detached, resuspended in Nano-Glo Live Cell Reagent (N2011, Promega) with furimazine (1:20 dilution), and seeded into white 96-well half area

plates. Luminescence was recorded at 37°C in a Flexstation 3 plate reader, with total luminescent signal used for NanoBiT assays and dual wavelength acquisition (460 and 535 nm) for NanoBRET assays. A 5-min baseline recording was followed by agonist addition and serial measurements over 30 min. Readings were taken every 30 s and normalized to well baseline, and then average vehicle-induced signal was subtracted to establish the agonist-induced effect. Areas under the curve (AUC) were calculated for each agonist concentration and fitted to four-parameter curves using GraphPad Prism 9.0.

Binding kinetic assay in primary islets

Islets were pre-incubated at 37°C with 1 μM exendin-4 in RPMI 1640 (with FBS and P/S) for 2 hours before imaging to saturate receptor binding to generate a negative control for TMR uptake, with exendin-4 maintained for the duration of imaging. These, alongside with untreated islets, were incubated with a metabolic inhibitor cocktail (20 mM 2-deoxyglucose and 10 mM NaN_3) to inhibit adenosine 5′-triphosphate–dependent endocytosis, as previously described (*70*), for 30 min before imaging. The metabolic inhibitors were maintained for the duration of the imaging.

Approximately 20 islets were then encased onto Matrigel in glass-bottom dishes and imaged in imaging medium (RPMI 1640 without phenol red, containing metabolic inhibitors) with a Zeiss LSM-780 inverted confocal laser-scanning microscope with a 10× objective from the FILM Facility at Imperial College London. TMR fluorescence was recorded every 2 s for 1 min baseline, followed by 100 nM exendin-4–TMR addition in imaging medium and recording for a further 15 min. Curve fitting to an exponential plateau was performed to calculate binding kinetic parameters in GraphPad Prism 9.0.

Generation of stable SNAP–GLP-1R–expressing β cell sublines

Five million INS-1 832/3 EV or g1-2 cells were seeded into 10-cm adherent dishes. Each dish was transfected with 9 μg of the SNAP–GLP-1R plasmid (Cisbio) using Lipofectamine 2000 according to the manufacturer's protocol. Forty-eight hours later, G418 (1 mg/ml) was added to each dish to select for SNAP–GLP-1R–positive cells. The surviving cells were allowed to proliferate. Once the 10-cm dishes reached >80% confluence, cells were labeled in suspension with SNAP-Surface 549 (New England Biolabs) for 30 min at 37°C and fluorescence-activated cell-sorted to select for SNAP–GLP-1R–expressing ones. Sorted cells were then cultured and maintained in G418 (1 mg/ml) to preserve SNAP–GLP-1R expression.

Immunoprecipitation assays

For NEDD4 coimmunoprecipitation, INS-1 832/3 EV or g1-2 SNAP-GLP-1R cells were transfected with the pCI HA NEDD4 construct (a gift from J. Massague, Addgene plasmid #27002) 24 hours before the experiment. Two million INS-1 832/3 EV or g1-2 SNAP-GLP-1R cells were seeded onto cell culture–treated six-well plates and allowed to attach overnight. The next day, the cells were treated with vehicle or 1 μM exendin-4 in RPMI 1640 (containing FBS, P/S, and additional Hepes, sodium pyruvate, and 2-mercaptoethanol) for 10 min at 37°C. Following stimulation, cells were washed 1× in ice-cold PBS and lysed in ice-cold 1× tris-buffered saline (TBS) [50 mM tris-HCl (pH 7.4) and 150 mM NaCl] supplemented with 1 mM EDTA, 1% Triton X-100, and protease and phosphatase inhibitor cocktails and lysates placed on a rocker for 15 min at 4°C. Next, lysates were incubated overnight under rotation at 4°C with ANTI-FLAG M2 Affinity Gel beads (Merck) to pull down FLAG-containing SNAP-GLP-1R, and pull-downs were performed according to the manufacturer's protocol.

Following the pull-down, the beads were resuspended in 1× TBS and 2× urea loading buffer [20 mM tris-HCl (pH 6.8), 5% SDS, 8 M urea, 100 mM dithiothreitol, and 0.02% bromophenol blue] 1:1 and incubated at 37°C for 10 min to separate pulled down proteins from beads. Samples were spun at 5000*g* for 30 s, and the supernatant was resolved in acrylamide gels, electroblotted onto polyvinylidene fluoride (PVDF) membranes, incubated with the indicated antibodies, and developed as detailed in the "Protein extraction and Western blotting" section. Specific band densities were quantified with ImageJ v1.53c. The details for the primary antibodies for ubiquitin and HA-tag and respective secondary antibodies are provided in Table 2.

Protein extraction and Western blotting

Protein extraction was performed by lysing samples with tris/NaCl/EDTA buffer [100 mM NaCl, 50 mM tris-HCl, 1 mM EDTA, and 0.1% BSA (pH 8.6)]. The samples were sonicated in a water bath sonicator 3× for 10 s. Urea loading buffer (2×) was added 1:1, and samples were incubated at 37°C for 10 min before resolving using SDS–polyacrylamide gel electrophoresis (10% acrylamide gels). Protein transfer to PVDF membranes (Immobilon-P, 0.45-μm pore size, IPVH00010, Merck) was achieved using a wet transfer system (Bio-Rad). The membranes were incubated with appropriate

Table 2. Primary and secondary antibodies used for Western blotting. Ab, antibody; RT, room temperature.

1° Ab	Manufacturer	Catalog no.	Dil.	Time, *T*°	2° Ab	Manufacturer	Catalog no.	Dil.	Time, *T*°
ERK 1/2	Cell Signaling Technology	9102S	1:1000	16 hours, 4°C	Rabbit	Abcam	ab205718	1:5000	1 hour, RT
pERK1/2	Cell Signaling Technology	9101S	1:1000	16 hours, 4°C	Rabbit	Abcam	ab205718	1:5000	1 hour, RT
CREB	Cell Signaling Technology	9197S	1:1000	16 hours, 4°C	Rabbit	Abcam	ab205718	1:5000	1 hour, RT
pCREB	Cell Signaling Technology	9198S	1:1000	16 hours, 4°C	Rabbit	Abcam	ab205718	1:5000	1 hour, RT
SNAP-tag	New England Biolabs	P9310S	1:500	16 hours, 4°C	Rabbit	Abcam	ab205718	1:5000	1 hour, RT
Ubiquitin	Santa Cruz Biotechnology	sc-8017	1:1000	16 hours, 4°C	Mouse	Abcam	Ab6728	1:5000	1 hour, RT
HA-tag	Sigma-Aldrich	H3663	1:1000	16 hours, 4°C	Mouse	Abcam	Ab6728	1:5000	1 hour, RT

primary and secondary antibodies listed in Table 2 in 5% milk and developed using the Clarity Western enhanced chemiluminescence substrate system (1705060, Bio-Rad) in a Xograph Compact X5 processor. Specific band densities were quantified using ImageJ v1.53c.

Cell labeling for confocal colocalization

INS-1 832/3 EV and g1-2 cells transiently expressing the SNAP-GLP-1R were labeled at 37°C with 1 μM SNAP-Surface 649 fluorescent substrate (S9159S, New England Biolabs) in full medium before treatment with 100 nM exendin-4 or vehicle for 3 hours. Five minutes before the end of the latter incubation period, 1 μM LysoTracker Red DND-99 (L7528, Thermo Fisher Scientific) was added. The cells were washed in PBS and fixed in 4% PFA, mounted in ProLong Diamond antifade reagent with 4′,6-diamidino-2-phenylindole (Life Technologies), and imaged by confocal microscopy with an Imperial College London NHLI FILM Zeiss LSM-780 inverted confocal laser-scanning microscope and a 63×/1.4 numerical aperture oil immersion objective equipped with Zen software (ZEN 2.3 SP1, black, 64-bit, Carl Zeiss). Images were analyzed using ImageJ v1.53c. The Coloc 2 plugin was used for colocalization analysis.

Time-lapse β-arrestin 2–GLP-1R colocalization by spinning disk microscopy

INS-1 832/3 cells stably expressing SNAP–GLP-1R were transiently transfected with a β-arrestin 2–GFP construct. Twenty-four hours after transfection, cells were seeded onto glass-bottom MatTek dishes and left to adhere overnight. Cells were labeled in full medium with SNAP-Surface 549 (New England Biolabs) for 30 min at 37°C and imaged in RPMI 1640 without phenol red in a Nikon Eclipse Ti spinning disk confocal microscope with an ORCA-Flash 4.0 camera (Hamamatsu) and Metamorph software (Molecular Devices). Time-lapse images of green and red fluorescences were acquired every 15 s for an initial 5-min baseline before the addition of 100 nM exendin-4 and further imaging for 10 min.

Electron microscopy

Islets extracted from chow diet-fed β cell β-arrestin 2 KO and control littermates were fixed in EM-grade 2% PFA + 2% glutaraldehyde mix overnight in 0.1 M cacodylate buffer and conventional EM was performed as described (*5*). Briefly, following fixation, islets were postfixed with osmium tetroxide, encased in 1% agarose, processed for EM, and embedded in Epon resin that was then polymerized at 60°C overnight. Seventy-nanometer-thick sections were cut with a diamond knife (DiATOME) in a Leica Ultracut UCT ultramicrotome before examination on an FEI Tecnai T12 Twin transmission EM. Images were acquired in a charge-coupled device camera (F216, TVIPS), and processed in ImageJ v1.53c.

Statistical analyses

For single-cell connectivity analysis, statistical differences were evaluated using paired or unpaired Student's *t* tests in MATLAB (MathWorks). All other data analyses and graph generation were performed with GraphPad Prism 9.0. The *n* numbers and statistical tests used are indicated in the corresponding figure legends. The number of replicates for comparisons (*n* numbers) represents biological replicates, defined as the number of mice per genotype for the in vivo experiments, or the number of biologically independent experiments performed from a separate batch of cells or islets extracted from separate mice for in vitro or ex vivo experiments, respectively. Technical replicates within biological replicates were averaged before statistical tests. Data are represented as means ± SEM, unless otherwise stated. The *P* value threshold for statistical significance was set at 0.05.

Supplementary Materials

This PDF file includes:
Figs. S1 to S7
Legend for movie S1

Other Supplementary Material for this manuscript includes the following:
Movie S1

View/request a protocol for this paper from *Bio-protocol*.

REFERENCES AND NOTES

1. M. A. Nauck, D. R. Quast, J. Wefers, J. J. Meier, GLP-1 receptor agonists in the treatment of type 2 diabetes–State-of-the-art. *Mol. Metab.* **46**, 101102 (2021).
2. K. Bettge, M. Kahle, M. S. Abd El Aziz, J. J. Meier, M. A. Nauck, Occurrence of nausea, vomiting and diarrhoea reported as adverse events in clinical trials studying glucagon-like peptide-1 receptor agonists: A systematic analysis of published clinical trials. *Diabetes Obes. Metab.* **19**, 336–347 (2017).
3. H. Zhang, E. Sturchler, J. Zhu, A. Nieto, P. A. Cistrone, J. Xie, L.-L. He, K. Yea, T. Jones, R. Turn, P. S. di Stefano, P. R. Griffin, P. E. Dawson, P. H. McDonald, R. A. Lerner, Autocrine selection of a GLP-1R G-protein biased agonist with potent antidiabetic effects. *Nat. Commun.* **6**, 8918 (2015).
4. B. Jones, The therapeutic potential of GLP-1 receptor biased agonism. *Br. J. Pharmacol.* **179**, 492–510 (2021).
5. B. Jones, T. Buenaventura, N. Kanda, P. Chabosseau, B. M. Owen, R. Scott, R. Goldin, N. Angkathunyakul, I. R. Corrêa Jr, D. Bosco, P. R. Johnson, L. Piemonti, P. Marchetti, A. M. J. Shapiro, B. J. Cochran, A. C. Hanyaloglu, A. Inoue, T. Tan, G. A. Rutter, A. Tomas, S. R. Bloom, Targeting GLP-1 receptor trafficking to improve agonist efficacy. *Nat. Commun.* **9**, 1602 (2018).
6. H. Kühn, U. Wilden, Deactivation of photoactivated rhodopsin by rhodopsin-kinase and arrestin. *J. Recept. Res.* **7**, 283–298 (1987).
7. M. J. Lohse, J. L. Benovic, J. Codina, M. G. Caron, R. J. Lefkowitz, β-Arrestin: A protein that regulates β-adrenergic receptor function. *Science* **248**, 1547–1550 (1990).
8. L. M. Luttrell, F. L. Roudabush, E. W. Choy, W. E. Miller, M. E. Field, K. L. Pierce, R. J. Lefkowitz, Activation and targeting of extracellular signal-regulated kinases by β-arrestin scaffolds. *Proc. Natl. Acad. Sci. U.S.A.* **98**, 2449–2454 (2001).
9. L. M. Luttrell, S. S. G. Ferguson, Y. Daaka, W. E. Miller, S. Maudsley, G. J. Della Rocca, F.-T. Lin, H. Kawakatsu, K. Owada, D. K. Luttrell, M. G. Caron, R. J. Lefkowitz, β-Arrestin-dependent formation of β_2 adrenergic receptor-Src protein kinase complexes. *Science* **283**, 655–661 (1999).
10. M. J. Lohse, C. Hoffmann, Arrestin interactions with G protein-coupled receptors. *Handb. Exp. Pharmacol.* **219**, 15–56 (2014).
11. S. S. Ferguson, W. E. Downey III, A. M. Colapietro, L. S. Barak, L. Ménard, M. G. Caron, Role of β-arrestin in mediating agonist-promoted G protein-coupled receptor internalization. *Science* **271**, 363–366 (1996).
12. S. A. Laporte, R. H. Oakley, J. A. Holt, L. S. Barak, M. G. Caron, The interaction of β-arrestin with the AP-2 adaptor is required for the clustering of β_2-adrenergic receptor into clathrin-coated pits. *J. Biol. Chem.* **275**, 23120–23126 (2000).
13. T. Buenaventura, S. Bitsi, W. E. Laughlin, T. Burgoyne, Z. Lyu, A. I. Oqua, H. Norman, E. R. McGlone, A. S. Klymchenko, I. R. Corrêa, A. Walker, A. Inoue, A. Hanyaloglu, J. Grimes, Z. Koszegi, D. Calebiro, G. A. Rutter, S. R. Bloom, B. Jones, A. Tomas, Agonist-induced membrane nanodomain clustering drives GLP-1 receptor responses in pancreatic beta cells. *PLOS Biol.* **17**, e3000097 (2019).
14. N. Sonoda, T. Imamura, T. Yoshizaki, J. L. Babendure, J.-C. Lu, J. M. Olefsky, β-Arrestin-1 mediates glucagon-like peptide-1 signaling to insulin secretion in cultured pancreatic β cells. *Proc. Natl. Acad. Sci. U.S.A.* **105**, 6614–6619 (2008).

15. A. Thompson, V. Kanamarlapudi, Agonist-induced internalisation of the glucagon-like peptide-1 receptor is mediated by the Gαq pathway. *Biochem. Pharmacol.* **93**, 72–84 (2015).
16. J. Quoyer, C. Longuet, C. Broca, N. Linck, S. Costes, E. Varin, J. Bockaert, G. Bertrand, S. Dalle, GLP-1 mediates antiapoptotic effect by phosphorylating bad through a β-arrestin 1-mediated ERK1/2 activation in pancreatic β-cells. *J. Biol. Chem.* **285**, 1989–2002 (2010).
17. B. Jones, E. R. McGlone, Z. Fang, P. Pickford, I. R. Corrêa Jr., A. Oishi, R. Jockers, A. Inoue, S. Kumar, F. Görlitz, C. Dunsby, P. M. W. French, G. A. Rutter, T. Tan, A. Tomas, S. R. Bloom, Genetic and biased agonist-mediated reductions in β-arrestin recruitment prolong cAMP signaling at glucagon family receptors. *J. Biol. Chem.* **296**, 100133 (2021).
18. G. Parruti, F. Peracchia, M. Sallese, G. Ambrosini, M. Masini, D. Rotilio, A. de Blasi, Molecular analysis of human beta-arrestin-1: Cloning, tissue distribution, and regulation of expression. Identification of two isoforms generated by alternative splicing. *J. Biol. Chem.* **268**, 9753–9761 (1993).
19. J. van Gastel, J. O. Hendrickx, H. Leysen, P. Santos-Otte, L. M. Luttrell, B. Martin, S. Maudsley, β-Arrestin based receptor signaling paradigms: Potential therapeutic targets for complex age-related disorders. *Front. Pharmacol.* **9**, 1369 (2018).
20. A. Srivastava, B. Gupta, C. Gupta, A. K. Shukla, Emerging functional divergence of β-arrestin isoforms in GPCR function. *Trends Endocrinol. Metab.* **26**, 628–642 (2015).
21. J. Wess, The two β-arrestins regulate distinct metabolic processes: Studies with novel mutant mouse models. *Int. J. Mol. Sci.* **23**, 495 (2022).
22. C. Benner, T. van der Meulen, E. Caceres, K. Tigyi, C. J. Donaldson, M. O. Huising, The transcriptional landscape of mouse beta cells compared to human beta cells reveals notable species differences in long non-coding RNA and protein-coding gene expression. *BMC Genomics* **15**, 620 (2014).
23. D. M. Blodgett, A. Nowosielska, S. Afik, S. Pechhold, A. J. Cura, N. J. Kennedy, S. Kim, A. Kucukural, R. J. Davis, S. C. Kent, D. L. Greiner, M. G. Garber, D. M. Harlan, P. dilorio, Novel observations from next-generation RNA sequencing of highly purified human adult and fetal islet cell subsets. *Diabetes* **64**, 3172–3181 (2015).
24. M. A. Ravier, M. Leduc, J. Richard, N. Linck, A. Varrault, N. Pirot, M. M. Roussel, J. Bockaert, S. Dalle, G. Bertrand, β-Arrestin2 plays a key role in the modulation of the pancreatic beta cell mass in mice. *Diabetologia* **57**, 532–541 (2014).
25. L. Zhu, J. Almaça, P. K. Dadi, H. Hong, W. Sakamoto, M. Rossi, R. J. Lee, N. C. Vierra, H. Lu, Y. Cui, S. M. McMillin, N. A. Perry, V. V. Gurevich, A. Lee, B. Kuo, R. D. Leapman, F. M. Matschinsky, N. M. Doliba, N. M. Urs, M. G. Caron, D. A. Jacobson, A. Caicedo, J. Wess, β-Arrestin-2 is an essential regulator of pancreatic β-cell function under physiological and pathophysiological conditions. *Nat. Commun.* **8**, 14295 (2017).
26. F. S. Willard, J. D. Douros, M. B. N. Gabe, A. D. Showalter, D. B. Wainscott, T. M. Suter, M. E. Capozzi, W. J. van der Velden, C. Stutsman, G. R. Cardona, S. Urva, P. J. Emmerson, J. J. Holst, D. A. D'Alessio, M. P. Coghlan, M. M. Rosenkilde, J. E. Campbell, K. W. Sloop, Tirzepatide is an imbalanced and biased dual GIP and GLP-1 receptor agonist. *JCI Insight* **5**, e140532 (2020).
27. A. Martinez-Sanchez, M. S. Nguyen-Tu, G. A. Rutter, DICER inactivation identifies pancreatic β-cell "disallowed" genes targeted by microRNAs. *Mol. Endocrinol.* **29**, 1067–1079 (2015).
28. T. J. Pullen, M. O. Huising, G. A. Rutter, Analysis of purified pancreatic islet beta and alpha cell transcriptomes reveals 11β-hydroxysteroid dehydrogenase (Hsd11b1) as a novel disallowed gene. *Front. Genet.* **8**, 41 (2017).
29. B. S. Muntean, S. Zucca, C. M. MacMullen, M. T. Dao, C. Johnston, H. Iwamoto, R. D. Blakely, R. L. Davis, K. A. Martemyanov, Interrogating the spatiotemporal landscape of neuromodulatory GPCR signaling by real-time imaging of cAMP in intact neurons and circuits. *Cell Rep.* **24**, 1081–1084 (2018).
30. P. H. Tewson, S. Martinka, N. C. Shaner, T. E. Hughes, A. M. Quinn, New DAG and cAMP sensors optimized for live-cell assays in automated laboratories. *J. Biomol. Screen.* **21**, 298–305 (2016).
31. S. J. Perry, G. S. Baillie, T. A. Kohout, I. McPhee, M. M. Magiera, K. L. Ang, W. E. Miller, A. J. McLean, M. Conti, M. D. Houslay, R. J. Lefkowitz, Targeting of cyclic AMP degradation to β_2-adrenergic receptors by β-arrestins. *Science* **298**, 834–836 (2002).
32. N. J. Pyne, B. L. Furman, Cyclic nucleotide phosphodiesterases in pancreatic islets. *Diabetologia* **46**, 1179–1189 (2003).
33. A. Kilanowska, A. Ziółkowska, Role of phosphodiesterase in the biology and pathology of diabetes. *Int. J. Mol. Sci.* **21**, 8244 (2020).
34. J. Zhu, E. Mix, B. Winblad, The antidepressant and antiinflammatory effects of rolipram in the central nervous system. *CNS Drug Rev.* **7**, 387–398 (2001).
35. A. Marzook, A. Tomas, B. Jones, The interplay of glucagon-like peptide-1 receptor trafficking and signalling in pancreatic beta cells. *Front Endocrinol (Lausanne)* **12**, 678055 (2021).
36. T. W. Chen, T. J. Wardill, Y. Sun, S. R. Pulver, S. L. Renninger, A. Baohan, E. R. Schreiter, R. A. Kerr, M. B. Orger, V. Jayaraman, L. L. Looger, K. Svoboda, D. S. Kim, Ultrasensitive fluorescent proteins for imaging neuronal activity. *Nature* **499**, 295–300 (2013).
37. V. Salem, L. D. Silva, K. Suba, E. Georgiadou, S. Neda Mousavy Gharavy, N. Akhtar, A. Martin-Alonso, D. C. A. Gaboriau, S. M. Rothery, T. Stylianides, G. Carrat, T. J. Pullen, S. P. Singh, D. J. Hodson, I. Leclerc, A. M. J. Shapiro, P. Marchetti, L. J. B. Briant, W. Distaso, N. Ninov, G. A. Rutter, Leader β-cells coordinate Ca^{2+} dynamics across pancreatic islets in vivo. *Nat. Metab.* **1**, 615–629 (2019).
38. E. Akalestou, K. Suba, L. Lopez-Noriega, E. Georgiadou, P. Chabosseau, A. Gallie, A. Wretlind, C. Legido-Quigley, I. Leclerc, V. Salem, G. A. Rutter, Intravital imaging of islet Ca^{2+} dynamics reveals enhanced β cell connectivity after bariatric surgery in mice. *Nat. Commun.* **12**, 5165 (2021).
39. M. Lucey, T. Ashik, A. Marzook, Y. Wang, J. Goulding, A. Oishi, J. Broichhagen, D. J. Hodson, J. Minnion, Y. Elani, R. Jockers, S. J. Briddon, S. R. Bloom, A. Tomas, B. Jones, Acylation of the incretin peptide exendin-4 directly impacts glucagon-like peptide-1 receptor signaling and trafficking. *Mol. Pharmacol.* **100**, 319–334 (2021).
40. E. R. McGlone, Y. Manchanda, B. Jones, P. Pickford, A. Inoue, D. Carling, S. R. Bloom, T. Tan, A. Tomas, Receptor activity-modifying protein 2 (RAMP2) alters glucagon receptor trafficking in hepatocytes with functional effects on receptor signalling. *Mol. Metab.* **53**, 101296 (2021).
41. R. Irannejad, J. C. Tomshine, J. R. Tomshine, M. Chevalier, J. P. Mahoney, J. Steyaert, S. G. F. Rasmussen, R. K. Sunahara, H. el-Samad, B. Huang, M. von Zastrow, Conformational biosensors reveal GPCR signalling from endosomes. *Nature* **495**, 534–538 (2013).
42. T. Buenaventura, N. Kanda, P. C. Douzenis, B. Jones, S. R. Bloom, P. Chabosseau, I. R. Corrêa Jr., D. Bosco, L. Piemonti, P. Marchetti, P. R. Johnson, A. M. J. Shapiro, G. A. Rutter, A. Tomas, A targeted RNAi screen identifies endocytic trafficking factors that control GLP-1 receptor signaling in pancreatic β-cells. *Diabetes* **67**, 385–399 (2018).
43. Z. W. Wang, X. Hu, M. Ye, M. Lin, M. Chu, X. Shen, NEDD4 E3 ligase: Functions and mechanism in human cancer. *Semin. Cancer Biol.* **67**, 92–101 (2020).
44. M. R. Dores, J. Trejo, Endo-lysosomal sorting of G-protein-coupled receptors by ubiquitin: Diverse pathways for G-protein-coupled receptor destruction and beyond. *Traffic* **20**, 101–109 (2019).
45. E. Rentzeperi, S. Pegiou, T. Koufakis, M. Grammatiki, K. Kotsa, Sex differences in response to treatment with glucagon-like peptide 1 receptor agonists: Opportunities for a tailored approach to diabetes and obesity care. *J. Pers. Med.* **12**, 454 (2022).
46. J. P. Frias, M. J. Davies, J. Rosenstock, F. C. Pérez Manghi, L. F. Landó, B. K. Bergman, B. Liu, X. Cui, K. Brown; for the SURPASS-2 Investigators, Tirzepatide versus semaglutide once weekly in patients with type 2 diabetes. *N. Engl. J. Med.* **385**, 503–515 (2021).
47. G. S. Baillie, A. Sood, I. McPhee, I. Gall, S. J. Perry, R. J. Lefkowitz, M. D. Houslay, β-Arrestin-mediated PDE4 cAMP phosphodiesterase recruitment regulates β-adrenoceptor switching from G_s to G_i. *Proc. Natl. Acad. Sci. U.S.A.* **100**, 940–945 (2003).
48. M. J. Willis, G. S. Baillie, Arrestin-dependent localization of phosphodiesterases. *Handb. Exp. Pharmacol.* **219**, 293–307 (2014).
49. S. J. Sanni, J. T. Hansen, M. M. Bonde, T. Speerschneider, G. L. Christensen, S. Munk, S. Gammeltoft, J. L. Hansen, β-Arrestin 1 and 2 stabilize the angiotensin II type I receptor in distinct high-affinity conformations. *Br. J. Pharmacol.* **161**, 150–161 (2010).
50. N. Saleh, G. Saladino, F. L. Gervasio, T. Clark, Investigating allosteric effects on the functional dynamics of β2-adrenergic ternary complexes with enhanced-sampling simulations. *Chem. Sci.* **8**, 4019–4026 (2017).
51. D. J. Hodson, R. K. Mitchell, E. A. Bellomo, G. Sun, L. Vinet, P. Meda, D. Li, W.-H. Li, M. Bugliani, P. Marchetti, D. Bosco, L. Piemonti, P. Johnson, S. J. Hughes, G. A. Rutter, Lipotoxicity disrupts incretin-regulated human β cell connectivity. *J. Clin. Invest.* **123**, 4182–4194 (2013).
52. N. Abdullah, M. Beg, D. Soares, J. S. Dittman, T. E. McGraw, Downregulation of a GPCR by β-arrestin2-mediated switch from an endosomal to a TGN recycling pathway. *Cell Rep.* **17**, 2966–2978 (2016).
53. A. Godbole, S. Lyga, M. J. Lohse, D. Calebiro, Internalized TSH receptors en route to the TGN induce local Gs-protein signaling and gene transcription. *Nat. Commun.* **8**, 443 (2017).
54. S. K. Shenoy, K. Xiao, V. Venkataramanan, P. M. Snyder, N. J. Freedman, A. M. Weissman, Nedd4 mediates agonist-dependent ubiquitination, lysosomal targeting, and degradation of the beta2-adrenergic receptor. *J. Biol. Chem.* **283**, 22166–22176 (2008).
55. C. E. Groer, C. L. Schmid, A. M. Jaeger, L. M. Bohn, Agonist-directed interactions with specific beta-arrestins determine mu-opioid receptor trafficking, ubiquitination, and dephosphorylation. *J. Biol. Chem.* **286**, 31731–31741 (2011).
56. N. Mosesso, M. K. Nagel, E. Isono, Ubiquitin recognition in endocytic trafficking - with or without ESCRT-0. *J. Cell Sci.* **132**, jcs232868 (2019).
57. S. Kaur, Y. Chen, S. K. Shenoy, Agonist-activated glucagon receptors are deubiquitinated at early endosomes by two distinct deubiquitinases to facilitate Rab4a-dependent recycling. *J. Biol. Chem.* **295**, 16630–16642 (2020).
58. K. Skieterska, P. Rondou, K. Van Craenenbroeck, Regulation of G protein-coupled receptors by ubiquitination. *Int. J. Mol. Sci.* **18**, 923 (2017).

59. V. Alonso, P. A. Friedman, Minireview: Ubiquitination-regulated G protein-coupled receptor signaling and trafficking. *Mol. Endocrinol.* **27**, 558–572 (2013).
60. S. Hammad, A. Othman, C. Meyer, A. Telfah, J. Lambert, B. Dewidar, J. Werle, Z. C. Nwosu, A. Mahli, C. Dormann, Y. Gao, K. Gould, M. Han, X. Yuan, M. Gogiashvili, R. Hergenröder, C. Hellerbrand, M. Thomas, M. P. Ebert, S. Amasheh, J. G. Hengstler, S. Dooley, Confounding influence of tamoxifen in mouse models of Cre recombinase-induced gene activity or modulation. *Arch. Toxicol.* **92**, 2549–2561 (2018).
61. X. Li, C. Clappier, I. Kleiter, R. Heuchel, Tamoxifen affects chronic pancreatitis-related fibrogenesis in an experimental mouse model: An effect beyond Cre recombination. *FEBS Open Bio* **9**, 1756–1768 (2019).
62. A. Fu, A. C.-H. Ng, C. Depatie, N. Wijesekara, Y. He, G.-S. Wang, N. Bardeesy, F. W. Scott, R. M. Touyz, M. B. Wheeler, R. A. Screaton, Loss of Lkb1 in adult beta cells increases beta cell mass and enhances glucose tolerance in mice. *Cell Metab.* **10**, 285–295 (2009).
63. P. Pickford, M. Lucey, Z. Fang, S. Bitsi, J. B. Serna, J. Broichhagen, D. J. Hodson, J. Minnion, G. A. Rutter, S. R. Bloom, A. Tomas, B. Jones, Signalling, trafficking and glucoregulatory properties of glucagon-like peptide-1 receptor agonists exendin-4 and lixisenatide. *Br. J. Pharmacol.* **177**, 3905–3923 (2020).
64. N. R. Johnston, R. K. Mitchell, E. Haythorne, M. P. Pessoa, F. Semplici, J. Ferrer, L. Piemonti, P. Marchetti, M. Bugliani, D. Bosco, E. Berishvili, P. Duncanson, M. Watkinson, J. Broichhagen, D. Trauner, G. A. Rutter, D. J. Hodson, Beta cell hubs dictate pancreatic islet responses to glucose. *Cell Metab.* **24**, 389–401 (2016).
65. A. Ertürk, K. Becker, N. Jährling, C. P. Mauch, C. D. Hojer, J. G. Egen, F. Hellal, F. Bradke, M. Sheng, H.-U. Dodt, Three-dimensional imaging of solvent-cleared organs using 3DISCO. *Nat. Protoc.* **7**, 1983–1995 (2012).
66. H. E. Hohmeier, H. Mulder, G. Chen, R. Henkel-Rieger, M. Prentki, C. B. Newgard, Isolation of INS-1-derived cell lines with robust ATP-sensitive K+ channel-dependent and -independent glucose-stimulated insulin secretion. *Diabetes* **49**, 424–430 (2000).
67. I. Miguel-Escalada, S. Bonàs-Guarch, I. Cebola, J. Ponsa-Cobas, J. Mendieta-Esteban, G. Atla, B. M. Javierre, D. M. Y. Rolando, I. Farabella, C. C. Morgan, J. García-Hurtado, A. Beucher, I. Morán, L. Pasquali, M. Ramos-Rodríguez, E. V. R. Appel, A. Linneberg, A. P. Gjesing, D. R. Witte, O. Pedersen, N. Grarup, P. Ravassard, D. Torrents, J. M. Mercader, L. Piemonti, T. Berney, E. J. P. de Koning, J. Kerr-Conte, F. Pattou, I. O. Fedko, L. Groop, I. Prokopenko, T. Hansen, M. A. Marti-Renom, P. Fraser, J. Ferrer, Human pancreatic islet three-dimensional chromatin architecture provides insights into the genetics of type 2 diabetes. *Nat. Genet.* **51**, 1137–1148 (2019).
68. K. J. Livak, T. D. Schmittgen, Analysis of relative gene expression data using real-time quantitative PCR and the $2^{-\Delta\Delta C_T}$ method. *Methods* **25**, 402–408 (2001).
69. J. B. Klarenbeek, J. Goedhart, M. A. Hink, T. W. J. Gadella, K. Jalink, A mTurquoise-based cAMP sensor for both FLIM and ratiometric read-out has improved dynamic range. *PLOS ONE* **6**, e19170 (2011).
70. Z. Fang, S. Chen, Y. Manchanda, S. Bitsi, P. Pickford, A. David, M. M. Shchepinova, I. R. Corrêa Jr, D. J. Hodson, J. Broichhagen, E. W. Tate, F. Reimann, V. Salem, G. A. Rutter, T. Tan, S. R. Bloom, A. Tomas, B. Jones, Ligand-specific factors influencing GLP-1 receptor post-endocytic trafficking and degradation in pancreatic beta cells. *Int. J. Mol. Sci.* **21**, 8404 (2020).

Acknowledgments: We thank S. Rothery from the NHLI FILM, Imperial College London, for technical assistance and help with data analysis; P. Chabosseau for providing a macro for β and α cell mass quantification in ImageJ; and S. Goudreau from ImmuPharma Group, France, for the synthesis of exendin-4–VT750. We additionally thank I. Leclerc, B. Owen, and A. Martinez-Sanchez for providing animal project licence access. **Funding:** This work was supported by MRC grant MR/R010676/1 (to A.T., B.J., and G.A.R.), UKRI COVID-19 Grant Extension Allocation (coA) (to AT.), and Society for Endocrinology Early Career Grant (to S.B.). **Author contributions:** Conceptualization: A.T. and B.J. Methodology: A.T., V.S., and B.J. Investigation: S.B., Y.M., L.E.E., A.I.O., N.M., B.H., K.S., and V.S. Visualization: S.B., V.S., and A.T. Supervision: A.T. and V.S. Writing—original draft: S.B. and A.T. Writing—review and editing: S.B., A.T., and B.J. Funding acquisition: A.T., G.A.R., and B.J. **Competing interests:** The authors declare that they have no competing interests. **Data and materials availability:** All data needed to evaluate the conclusions in the paper are present in the paper and/or the Supplementary Materials.

Submitted 11 November 2022
Accepted 4 April 2023
Published 3 May 2023
10.1126/sciadv.adf7737
